# Loss of *Sirt2* increases and prolongs a caerulein-induced pancreatitis permissive phenotype and induces spontaneous oncogenic *Kras* mutations in mice

**DOI:** 10.1038/s41598-018-34792-y

**Published:** 2018-11-07

**Authors:** Songhua Quan, Daniel R. Principe, Angela E. Dean, Seong-Hoon Park, Paul J. Grippo, David Gius, Nobuo Horikoshi

**Affiliations:** 1 Department of Radiation Oncology, Northwestern University Feinberg School of Medicine, Chicago, IL USA; 2 Department of Pharmacology, Robert Lurie Cancer Center, Northwestern University Feinberg School of Medicine, Chicago, IL USA; 3 Department of Medicine, University of Illinois at Chicago, Chicago, IL USA; 4 Present Address: Department of General and Applied Toxicology, Genetic Toxicology Research Group, Korea Institute of Toxicology (KIT), Daejeon South Korea

## Abstract

Mice lacking *Sirt2* spontaneously develop tumors in multiple organs, as well as when expressed in combination with oncogenic *Kras*^*G12D*^, leading to pancreatic tumors. Here, we report that after caerulein-induced pancreatitis, *Sirt2*-deficient mice exhibited an increased inflammatory phenotype and delayed pancreatic tissue recovery. Seven days post injury, the pancreas of *Sirt2*^−/−^ mice display active inflammation, whereas wild-type mice had mostly recovered. In addition, the pancreas from the *Sirt2*^−/−^ mice exhibited extensive tissue fibrosis, which was still present at six weeks after exposure. The mice lacking *Sirt2* also demonstrated an enhanced whole body pro-inflammatory phenotype that was most obvious with increasing age. Importantly, an accumulation of a cell population with spontaneous cancerous *Kras*^*G12D*^ mutations was observed in the *Sirt2*^−/−^ mice that is enhanced in the recovering pancreas after exposure to caerulein. Finally, transcriptome analysis of the pancreas of the *Sirt2*^−/−^ mice exhibited a pro-inflammatory genomic signature. These results suggest that loss of *Sirt2*, as well as increased age, enhanced the immune response to pancreatic injury and induced an inflammatory phenotype permissive for the accumulation of cells carrying oncogenic *Kras* mutations.

## Introduction

Sirtuin genes are the human and murine homologs of the *S. cerevisiae Sir2* gene that have been shown to direct both metabolism and lifespan^1^. In addition, sirtuins play a role in longevity by directing critical acetylome signaling networks in response to caloric restriction^2^. Sirtuin 2 (SIRT2) is a member of sirtuin family of NAD^+^-dependent deacetylases that are linked to multiple biological and pathological processes, such as the cell cycle, DNA repair, metabolic homeostasis, genomic integrity, and tumorigenesis. Originally, SIRT2 was observed to regulate the cell cycle^3^ through the deacetylation of H4K16ac^4^ but later was shown also to function in metabolic processes by inhibiting adipogenesis by deacetylating FOXO1 and facilitating lipolysis by repressing PPARγ activity^5^. SIRT2 also directs other physiological processes, including gluconeogenesis, insulin sensitivity, and fatty acid oxidation^6–8^, and is critical for insulin-dependent AKT activation, suggesting that the inhibition of SIRT2 may have an impact on cancer growth^9^.

We have shown that mice lacking *Sirt2* develop tumors in multiple organs, including pancreatic ductal adenocarcinomas (PDAC)^10^, and interestingly, the combination of *Sirt2* deletion with an oncogenic *Kras*^*G12D*^ mutation induces PDAC at three months^11^. In this regard, it has also been shown that KRAS is a SIRT2 downstream deacetylation target that directs enzyme activity^12^. Ninety-five percent of patients diagnosed with PDAC have tumors with a *KRAS* mutation. However, the percentage of oncogenic mutant KRAS in healthy organs, including the pancreas, are at rates far exceeding the incidence of cancer development. Furthermore, KRAS GTPase activity is higher in cells derived from mutant *Kras*-driven PDAC as compared to non-transformed pancreatic cells expressing the same mutant *Kras*^13^, suggesting that other events are necessary for complete somatic cell dedifferentiation and/or transformation^14,15^.

Pancreatitis is initiated by the premature activation of digestive enzymes that cause auto-digestion of the gland^16–18^ and commonly is caused by gallstones, excessive alcohol consumption, smoking, medications, and blunt abdomen trauma^19^. However, severe and recurrent episodes can develop chronic pancreatitis, leading to permanent inflammation of the pancreas and an increased risk of pancreatic cancer. Caerulein-induced pancreatitis is a well-characterized rodent model that mimics clinical pancreatitis^16,18,20–22^. Injury of the pancreas induced by caerulein results in infiltration of inflammatory immune cells, edema, and destruction of a large portion of the pancreatic parenchyma^20,21,23^. Despite the massive inflammatory response and tissue destruction, the murine pancreas can histologically regenerate and recover after exposure^24,25^, often within one week^20,23,26–28^.

In this regard, a recent study has revealed the role of SIRT2 in inflammatory responses, in which SIRT2 deacetylates the NF-κB subunit p65, resulting in a decrease of NF-κB-dependent gene expression^29^. More recently, loss of *Sirt2* increases the severity of experimental colitis by modulation of macrophage polarization^30^ and collagen-induced arthritis with a concomitant increase in NF-κB acetylation^31^. In addition, agents that inhibit SIRT2 aggravates experimental traumatic brain injury by increasing acetylation and nuclear translocation of NF-κB p65^32^. Finally, since 2017, there are now over twenty manuscripts that describe, at least in some part, a mechanistic connection between SIRT2, protein acetylation, and a potential link to inflammatory pathways. Thus, it seems reasonable to propose that these studies provide evidence for an anti-inflammatory effect of SIRT2.

As mentioned, we have shown that *Sirt2* knockout mice with a *KRAS*^*G12D*^ mutation background develop pancreatic cancer, suggesting *Sirt2* knockout is susceptible for pancreatic cancer^11^. Also, SIRT2 has been shown to be involved in the inflammatory response^29–31^, and pancreatic inflammation is a key issue in pancreatic cancer incidents in humans. Since *Kras* mutations are found in more than 95% of pancreatic cancers in human, we hypothesized that sustained inflammation due to the lack of SIRT2 activity accumulates a cell population carrying *Kras* mutations that originally existed in the pancreas through the disruption-regeneration cycles during pancreatitis. Here, we report that after caerulein-induced pancreatitis, *Sirt2*-deficient mice exhibited an increased inflammatory phenotype and delayed recovery. In addition, aged *Sirt2*^−/−^ mice develop a pro-inflammatory permissive phenotype. Interestingly, we found that the oncogenic mutations in *Kras* accumulation was drastically accelerated in pancreatic cells of *Sirt2*^−/−^ mice with caerulein-induced acute pancreatitis. Our results indicate that *Sirt2* deficiency increases inflammation infiltration during caerulein-induced acute pancreatitis and strongly impairs the recovery from pancreatic tissue injury, as well as leads to the accumulation of oncogenic *Kra*s mutations in the pancreas.

## Results

### *Sirt2* deficiency impairs pancreatic regeneration

**Figure 1:**
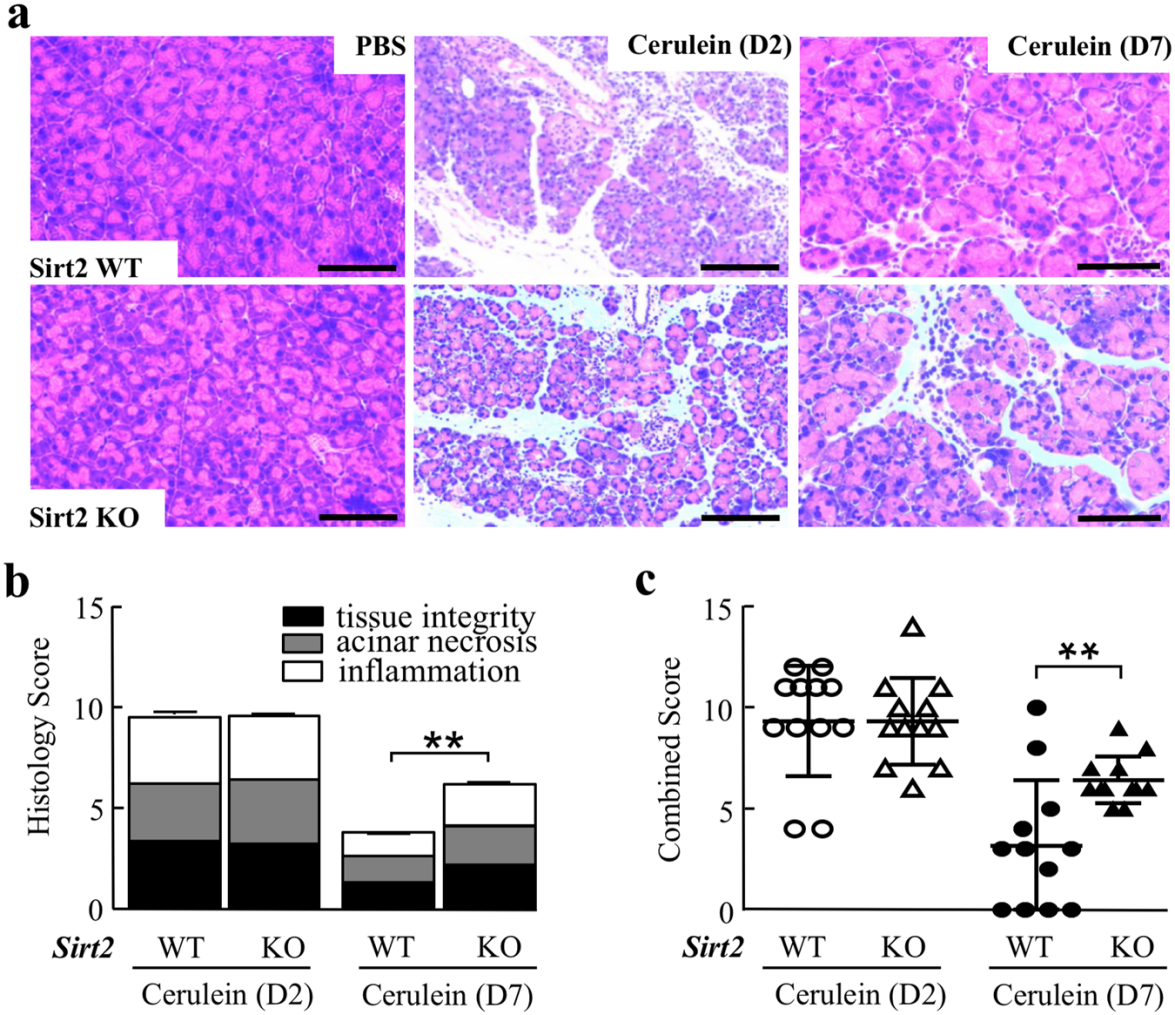
Mice lacking *Sirt2* exposed to caerulein exhibit impaired pancreatic regeneration. **(a)** Wild-type (WT) and *Sirt2*^−/−^ (KO) mice had 8 hourly intraperitoneal (i.p.) injections with PBS or caerulein (100 μg/ml), and the pancreas was harvested on day 2 (D2) or day 7 (D7) post-injection. H&E staining was done for all mice, and representative images are shown. n = 5–10 mice per group. Bars indicate 100 µm. **(b)** Individual scoring of pancreatic tissue integrity, acinar necrosis, and inflammation parameters from wild-type and *Sirt2*^−/−^ mice used in Fig. 1a. **(c)** Combined scoring of pancreatic tissue integrity for the mice treated without or with caerulein and harvested at D2 and D7. The scoring system used is shown in the Supplemental Section, Table 1. (n = 5–10, *p < 0.05, **p < 0.01).

It has been previously shown that loss of *Sirt2* accelerates the oncogenic properties of oncogenic *Kras*^*G12D*^, and these tumors exhibited an increase in trichrome staining suggesting a potential pro-inflammatory environment in pancreatic cells lacking *Sirt2*^11^. To address this question, we used a well-established model to induce pancreatitis by 8 hourly intraperitoneal (i.p.) injections of 100 μg/kg caerulein. After two days post injection, wild-type and *Sirt2*^−/−^ mice exposed to caerulein were sacrificed and both showed similar patterns of moderately severe histological damage (Fig. 1a), and scores of tissue integrity, acinar cell necrosis, as well as inflammatory cell infiltration (Fig. 1b,c) (Supplemental Section, Table S1, for histological scoring criteria). In contrast, wild-type mice pancreas appear to mostly recover at day 7 post injection; however, pancreatic regeneration was severely impaired in *Sirt2*^−/−^ mice, and the inflammation score remained ~70% of day 2 (Fig. 1a–c). Taken together, these results suggest that loss of *Sirt2* led to delayed pancreas tissue recovery after caerulein-induced acute pancreatitis, suggesting SIRT2 may play role in pancreatic tissue regeneration.

### Loss of *Sirt2* deficiency leads to inflammatory cell infiltration following caerulein exposure

**Figure 2:**
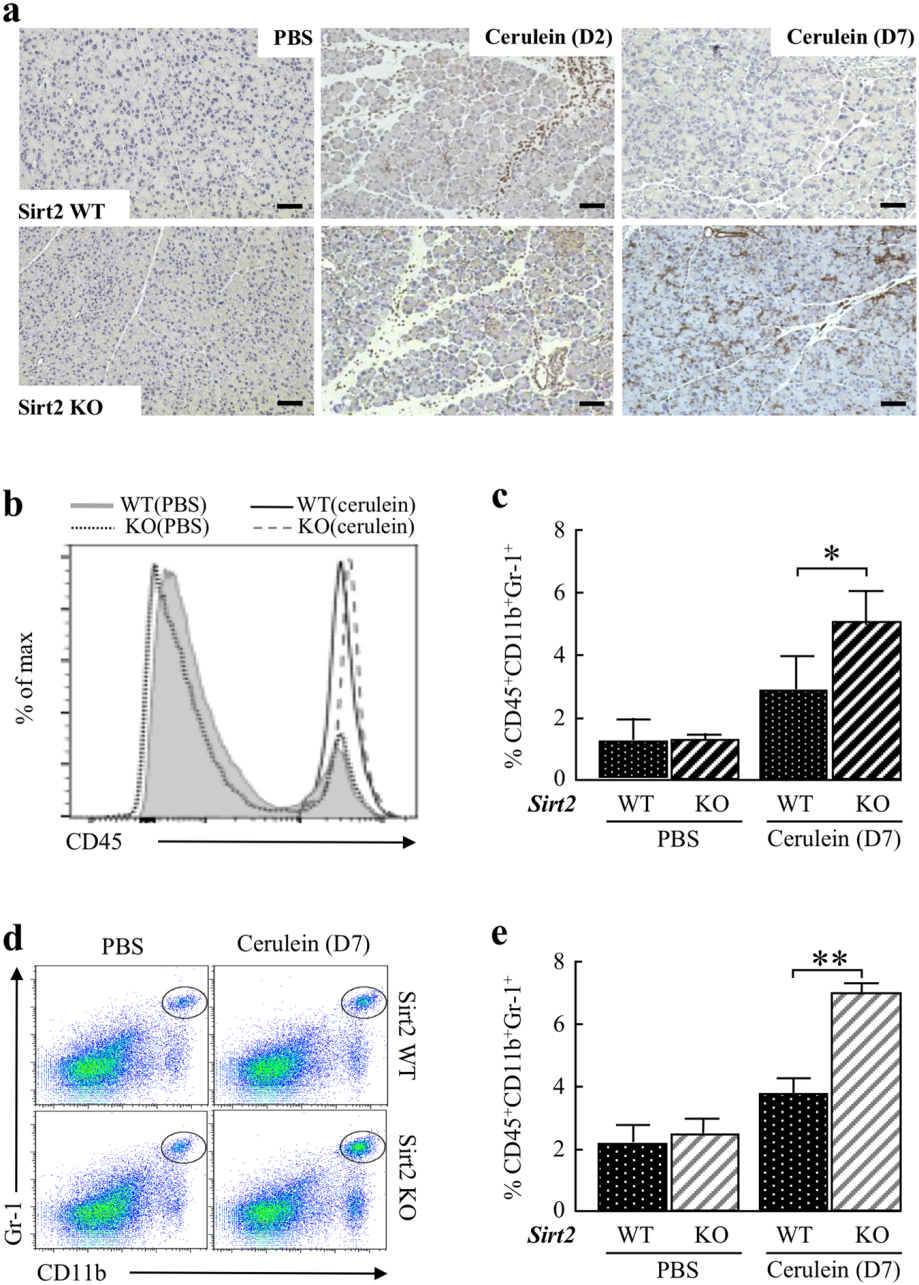
Deletion of *Sirt2* increases the macrophage-predominant inflammatory response. **(a)** Wild-type (WT) and *Sirt2*^−/−^ (KO) mice were i.p. injected with PBS or caerulein, and the pancreas was harvested on day 2 (D2) or day 7 (D7) post-injection. Panels showed representative immuno-histochemical staining of a macrophage maker F4/80. Bars indicate 100 µm. **(b,c)** Pancreas from wild-type and *Sirt2*^−/−^ mice treated with PBS or caerulein at D7 were harvested, digested, and single cells were isolated and stained with anti-CD45, anti-CD11b, and anti-Gr-1 antibodies. **(d)** FACS plots characterized the expression of CD45^+^ cells in wild-type, and *Sirt2*^−/−^ mice at D7. Wild-type and *Sirt2*^−/−^ mice were i.p. injected with PBS or caerulein, and the spleen was removed at D7. Splenic cells were isolated and stained with anti-CD45, anti-CD11b, and anti-Gr-1 antibodies. Scatterplots showed the population of CD45^+^CD11b^+^Gr1^+^ myeloid cells in wild-type and *Sirt2*^−/−^ mice at D7 (circled). **(e)** Graph showing the percentage of CD45^+^CD11b^+^Gr1^+^ myeloid cells from wild-type and *Sirt2*^−/−^ at D7. (n = 5, *p < 0.05, **p < 0.01). Bar graphs indicated mean ± standard error of the mean (s.e.m).

To determine whether loss of *Sirt2* affects the inflammatory cell infiltration in pancreatic tissue during a course of acute caerulein exposure, we assessed the IHC staining of a macrophage maker F4/80. Wild-type and *Sirt2*^−/−^ mice displayed a similar F4/80^+^ IHC staining in macrophages infiltrating into the pancreatic tissue at day 2 (Fig. 2a, left and middle panels), as compared to PBS-injected controls. In contrast, F4/80^+^ staining macrophage infiltration was still observed in *Sirt2*^−/−^ mice at day 7 (Fig. 2a, right panels).

**Figure 3:**
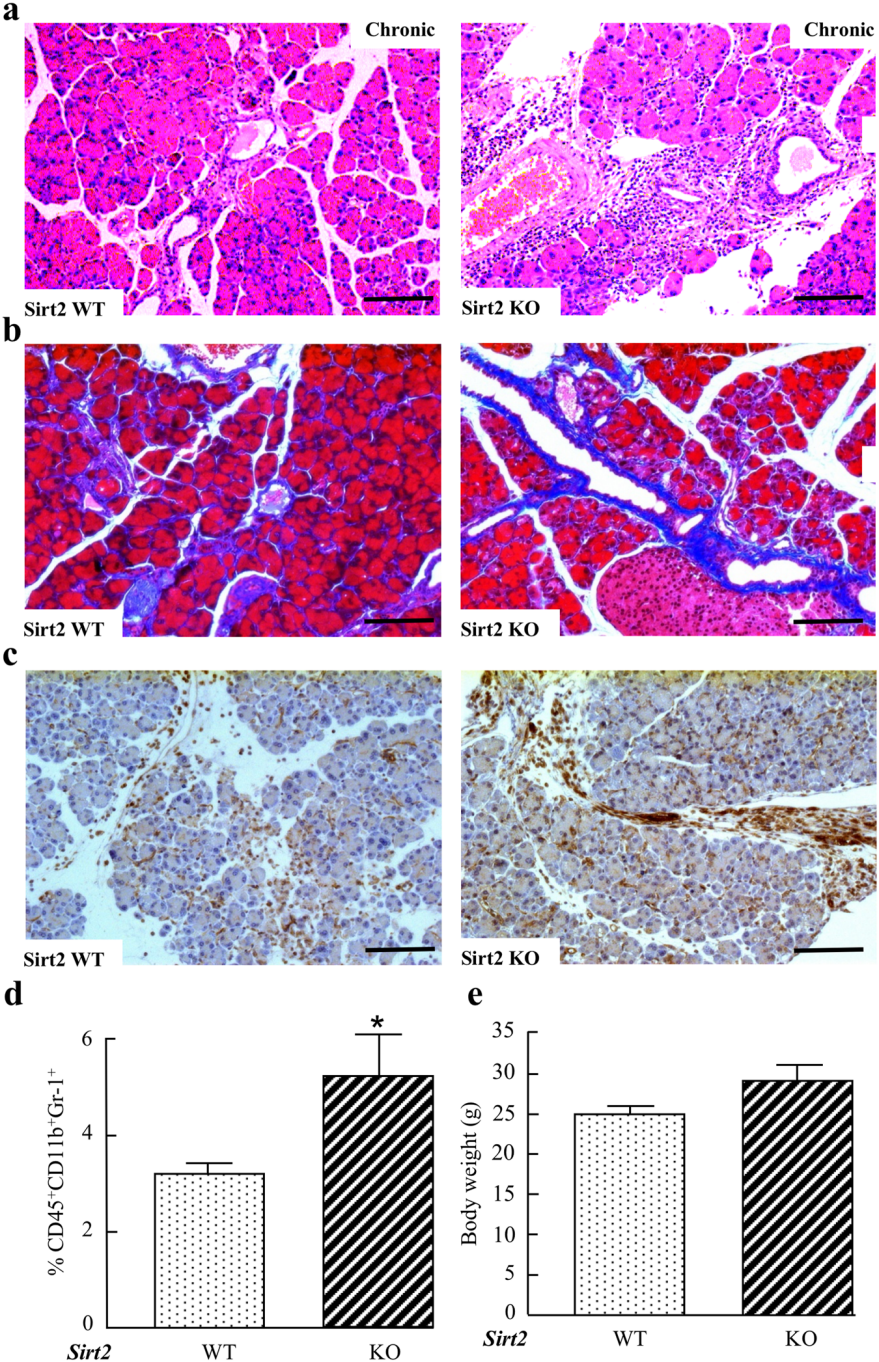
Characterization of wild-type and *Sirt2* knockout mice pancreas at 6 weeks after caerulein-induced chronic pancreatitis. **(a)** Wild-type (WT) and *Sirt2*^−/−^ (KO) mice had 6 hourly intraperitoneal (i.p.) injections with caerulein (50 μg/ml) once a week for 6 weeks. H&E staining was done for all mice, and representative images are shown. n = 5 mice per group. Bars indicate 100 µm. **(b)** Wild-type (WT) and *Sirt2*^−/−^ (KO) mice pancreas with caerulein-induced chronic pancreatitis at 6 weeks were analyzed by Masson’s Trichrome staining. Representative images are shown. Bars indicate 100 µm. **(c)** Wild-type (WT) and *Sirt2*^−/−^ (KO) mice were i.p. injected with caerulein, and the pancreas was harvested at 6 weeks post-injection. Panels showed representative immuno-histochemical staining of a macrophage maker F4/80. Bars indicate 100 µm. **(d)** Measurement of pancreas infiltrated macrophages in wild type and *Sirt2*^−/−^ mice at 6 weeks after caerulein-induced chronic pancreatitis. **(e)** Body weight of mice at 6 weeks after caerulein-induced chronic pancreatitis.

The local pancreatic inflammatory response was assessed in isolated pancreas in *Sirt2*^−/−^ mice and wild-type mice at day 7 post injection (Fig. 2b,c). CD45^+^ macrophages were increased by caerulein-induced acute pancreatitis in the pancreas from both wild type and *Sirt2*^−/−^ mice, and the induced levels for both were maintained at day 7 to a similar extent. However, the myeloid-derived suppressor cell (MDSC) population (CD45^+^CD11b^+^Gr-1^+^) was significantly higher in pancreas from *Sirt2*^−/−^ mice as observed in spleen confirming sustained pancreatitis at day 7. Next, we examined whether the whole body CD45^+^CD11b^+^Gr-1^+^ have been changed in the spleen following caerulein injection. The increase of the CD45^+^CD11b^+^Gr-1^+^ population positively correlates with inflammation and being cancer bearing. A high proportion of CD45^+^CD11b^+^Gr-1^+^ cells were observed in the spleen of *Sirt2*^−/−^ mice compared to wild-type mice at D7 after the last injection (Fig. 2d,e). IFN-γ producing CD4^+^ T cells and serum level of IFN-γ also remained high in *Sirt2*^−/−^ mice at day 7 (Supplemental Section, Fig. S1). Pancreatic tissue damage, as well as macrophage infiltration, was also abnormal, as compared to control mice, up to at least 21 days after the induction of pancreatitis (Supplemental Section, Figs S2 and S3). Since SIRT2 regulates NF-κB activities^29^, we assessed the NF- κB activity in pancreas from wild-type and *Sirt2*^−/−^ mice at day 7. The expression levels of NF-κB target genes, *Pdgfra* (Platelet Derived Growth Factor Receptor Alpha), *Fcer1g* (Fc Fragment Of IgE Receptor Ig), and *Ccl2* (C-C Motif Chemokine Ligand 2) were determined by RT-qPCR and showed sustained NF-κB activity at day 7 in *Sirt2*^−/−^ mice (Supplemental Section, Fig. S4), indicating the sustained active immunological response in pancreas from *Sirt2*^−/−^ mice at day 7. Finally, when a chronic pancreatitis model was used, via injection of a lower concentration of caerulein into mice (50 μg/kg, 6 hourly injections once a week for 6 weeks), the pancreas from the *Sirt2*^−/−^ mice also exhibited more intense tissue damage (Fig. 3a,b) and macrophage infiltration (Fig. 3c,d) although there is no difference in body weight (Fig. 3e). The results of these *in vivo* experiments suggest that loss of *Sirt2* prolonged the whole body inflammatory response by caerulein-induced acute and chronic pancreatitis.

### Effect of *Sirt2* deficiency on immune response by age

*Sirt2*-deficient mice showed prolonged inflammation in caerulein-induced acute pancreatitis, suggesting that these mice may have intrinsic problems with their immune response. Our previous finding that *Sirt2*-deficient mice developed pancreatic cancer with an oncogenic mutant *Kras* background^11^ also supports our notion since tumor development is closely linked with the increasing age partly due to the impaired immune system integrity^33^.

**Figure 4:**
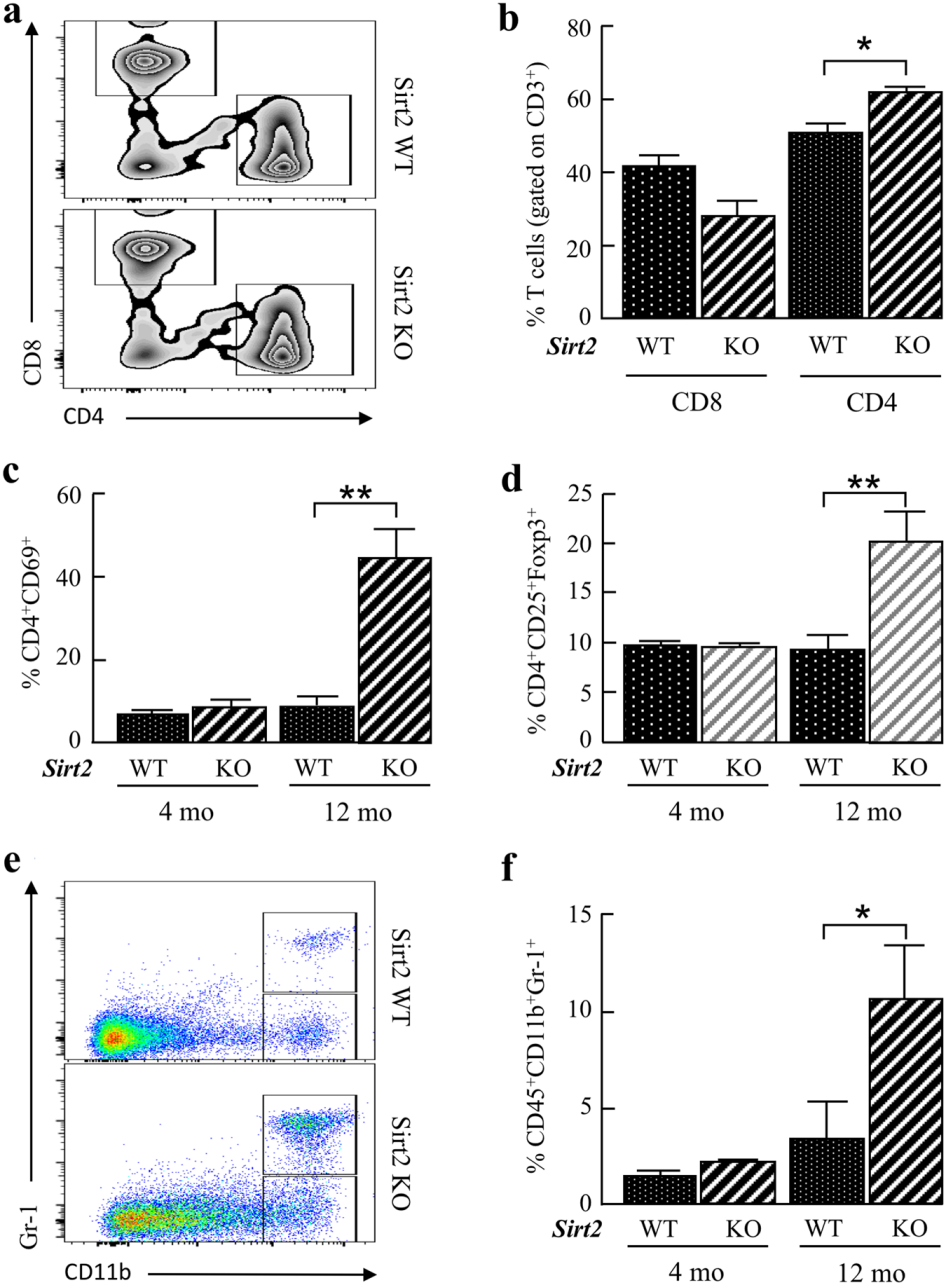
Mice lacking *Sirt2* exhibit age-dependent difference in T cell activity, as well as macrophage infiltration. **(a)** The spleens from 12-month old wild-type (WT) and *Sirt2*^−/−^ (KO) mice were harvested, and splenocytes were stained with anti-CD3, anti-CD8, and anti-CD4 antibodies. FACS plots showed the populations of CD3^+^CD8^+^ T cells and CD3^+^CD4^+^ T cells from wild-type or *Sirt2*^−/−^ mice. **(b)** Quantitative analysis of the cytotoxic (CD3^+^CD8^+^) and helper (CD3^+^CD4^+)^ T cells from wild-type and *Sirt2*^−/−^ mice from Fig. 3a. **(c)** The spleens from age-matched (4 and 12 month) wild-type (WT) and *Sirt2*^−/−^ (KO) mice were harvested, and splenocytes were stained with anti-CD3, anti-CD4, and anti-CD69 antibodies. The graph shows the percentage of CD3^+^CD4^+^CD69^+^ active T cells from wild-type or *Sirt2*^−/−^ mice at 4 and 12 months of age. **(d)** The spleens from age-matched wild-type (WT) and *Sirt2*^−/−^ (KO) mice were harvested, and splenocytes were stained with anti-CD4, anti-CD25, and anti-Foxp3 antibodies. The graph indicates the percentage of CD4^+^CD25^+^Foxp3^+^ regulatory T cells from wild-type or *Sirt2*^−/−^ mice at 4 and 12 months of age. **(e)** The spleens from 12-month old wild-type (WT) and *Sirt2*^−/−^ (KO) mice were harvested, and splenocytes were stained with anti-CD45, anti-CD11b, and anti-Gr-1 antibodies. Scatterplots showed the population of CD45^+^CD11b^+^Gr-1^+^ cells. **(f)** Quantitative analysis of the CD45^+^CD11b^+^Gr-1^+^ cells from wild-type and *Sirt2*^−/−^ mice at 12-month old from Fig. 3e (n = 4, *p < 0.05, **p < 0.01).

To study the effects of *Sirt2* deficiency on the systemic immune response, age-matched mice were analyzed by flow cytometry to determine the age-related changes in immune cell proportions, such as CD4^+^ T cells, CD8^+^ T cells, active T cells (CD4^+^CD69^+^), Tregs (CD4^+^CD25^+^Foxp3^+^), and MDSCs (CD45^+^CD11b^+^Gr-1^+^) in the spleen. Compared to the wild-type mice, the *Sirt2*^−/−^ mice exhibited an increase in the percentage of the total CD4^+^ T cells and a decrease in the percentage of total CD8^+^ T cells in the spleen of 12-month old mice (Fig. 4a,b). In addition, the *Sirt2*^−/−^ mice also exhibited an increased proportion of activated T cells (CD4^+^CD69^+^, Fig. 4c) and Tregs (CD4^+^CD25^+^Foxp3^+^, Fig. 4d) in the spleen of 12-month old mice, but not in 4-month old mice. Notably, *Sirt2* deletion leads to a higher proportion of inflammatory CD45^+^CD11b^+^Gr-1^+^ cells and CD45^+^CD11b^+^Gr-1^−^ cells, such as red pulp macrophages in the spleen of 12-month old, but not 4-month old mice (Fig. 4e,f). These results suggest that loss of *Sirt2* shows enhanced inflammatory responses during aging, even without exposing an agent that induces an inflammation response.

### Loss of *Sirt2* leads to elevated serum amylase and prolonged fibrosis

**Figure 5:**
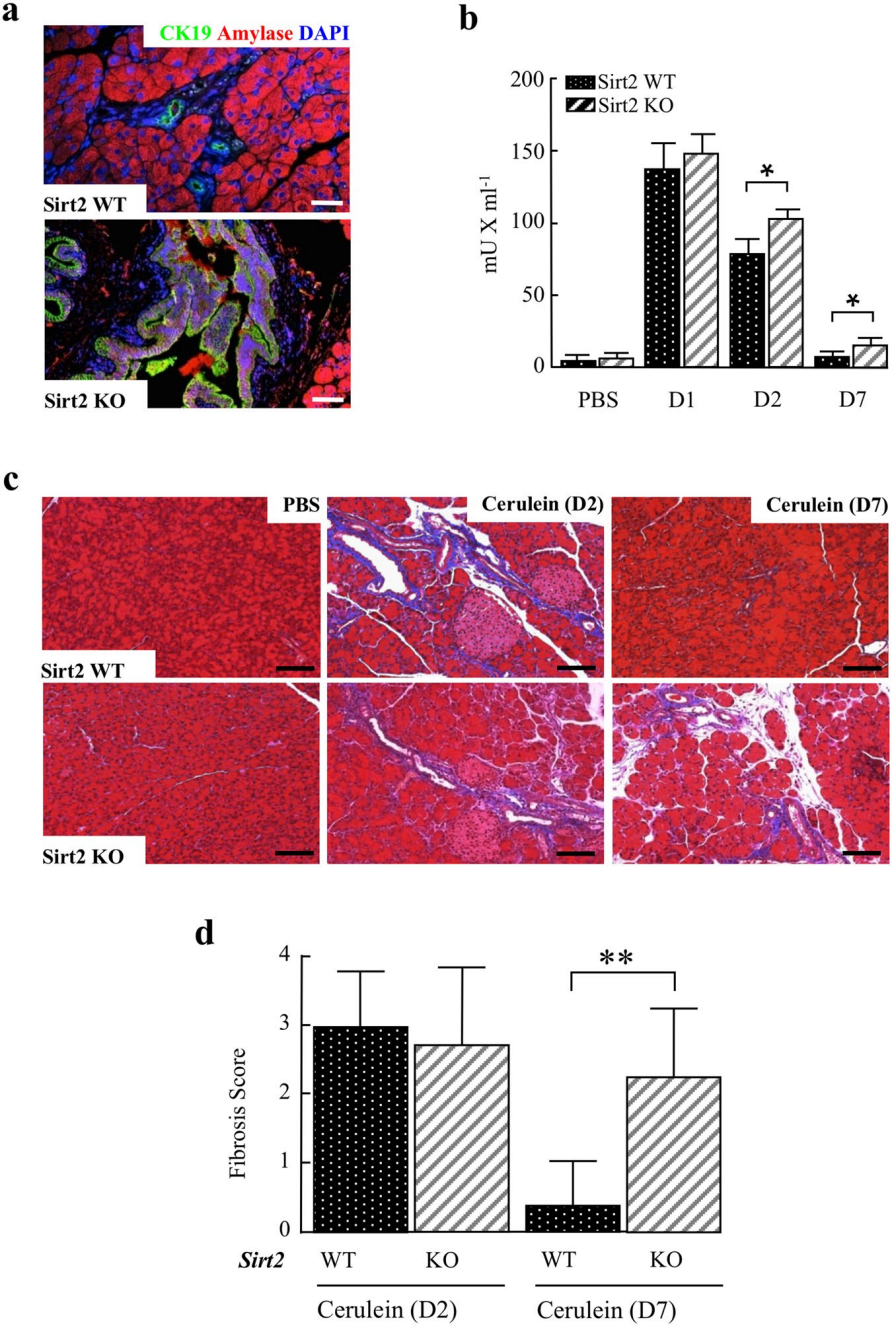
*Sirt2*-deficiency leads to the progression of acute pancreatitis to pre-invasive neoplasms. **(a)** Wild-type and *Sirt2*^−/−^ mice were i.p. injected with caerulein, and pancreatic tissue was harvested at D7. Immuno-fluorescent staining was performed with anti-CK19 (green), and pancreatic anti-amylase (red). Colocalization of CK19 and pancreatic amylase is shown. Bars indicate 100 µm. **(b)** Wild-type and *Sirt2*^−/−^ mice were i.p. injected with PBS or caerulein, and serum was collected prior to injection and on days 1, 2, and 7 post injection. Serum amylase was measured with an amylase assay kit purchased from Abcam. (n = 5–10, p < 0.05, **p < 0.01). **(c**) Wild-type and *Sirt2*^−/−^ mice pancreas with caerulein-induced pancreatitis on day 2 (D2) and day 7 (D7) were analyzed by Masson’s Trichrome staining. Blue staining indicates collagen fibers. Note that the pancreas from the *Sirt2*^−/−^ mouse still shows histological damage at day 7, whereas the wild type mouse pancreas shows remarkable recovery. Representative images are shown. **(d)** Quantitative analysis of stromal fibrosis in wild-type and *Sirt2*^−/−^ mice pancreas from Fig. 4a was performed by scoring the intensity of stromal fibrosis, as determined by the percentage of blue stained areas. (n = 5, *p < 0.05, **p < 0.01).

To monitor tissue damage and regeneration of the pancreas in a caerulein-induced model for acute pancreatitis, the wild-type and *Sirt2*^−/−^ mice pancreas were stained for CK19, amylase, and DAPI. Wild-type mice, 7 days after exposure, showed a predominate amylase staining pattern, an acinar marker consistent with tissue recovery (Fig. 5a) while in contrast, the *Sirt2*^−/−^ mice exhibited significant CK19 staining, a ductal marker indicating the presence of tissue damage (Fig. 5a). In addition, the levels of serum amylase in the *Sirt2*^−/−^ mice were also significantly higher, as compared to wild-type mice, at day 2 and 7 post caerulein injection, indicating sustained tissue and prolonged damage has occurred in mice lacking *Sirt2* (Fig. 5b).

Pancreatic fibrosis is a characteristic histopathological feature of chronic pancreatitis due to tissue injury. Pancreatic fibrosis, as measured by Masson’s Trichrome staining, was similar in both the wild-type and *Sirt2*^−/−^ mice on day 2 post injection, as seen by the blue stained areas (Fig. 5c, middle panel). The expression level for α-smooth muscle actin (α-SMA) as a fibrosis marker was also similar between wild-type and *Sirt2*^−/−^ mice (Supplemental Section, Fig. S5). At day 7 post injection, the pancreas from wild-type mice were mostly recovered, and only sporadic blue sections were found (Fig. 5c, right-upper panel, Fig. 5d). In contrast, pancreas from *Sirt2*^−/−^ mice still exhibited significant areas of blue staining (Fig. 5c, right-bottom panel, Fig. 5d), suggesting the continued presence of pancreatic fibrosis. Recovery from caerulein-induced pancreatitis is evident at day 7 from pancreas/body weight ratio in wild-type mice (Supplemental Section, Fig. S6a), as well as continuous regeneration/proliferation by Ki67 stain in *Sirt2*^−/−^ mice (Supplemental Section, Fig. S6b). To assess the elimination mechanism of cells at day 7, we determined p53 expression by RT-qPCR (Supplemental Section, Fig. S7a) and immunohistochemistry (Supplemental Section, Fig. S7b) and found that there was no difference in p53 expression level between wild-type and *Sirt2*^−/−^ mice at day 7, suggesting a minor contribution of p53-dependent apoptotic pathways.

### Enhanced accumulation of spontaneous Kras mutations in *Sirt2* knockout mice at 7 days after caerulein-induced pancreatitis

Pancreatic cell *KRAS* mutations, which commonly occur at amino acid position 12, glycine (G), to valine (V) or aspartic acid (D), are a very early precancerous event^34–36^ and are also found in normal cell populations^15,37^. In addition, pancreatic fibrosis is also a high-risk factor for PDAC^38^. Taken together, we hypothesized that the pro-inflammatory/pro-fibrotic permissive phenotype observed in mice lacking *Sirt2* may select for cell populations that exhibit growth advantages, such as carrying an oncogenic *Kras* mutation. Indeed, a cross relationship between inflammation and *KRAS* mutations has been suggested previously^2,39–42^.

**Figure 6:**
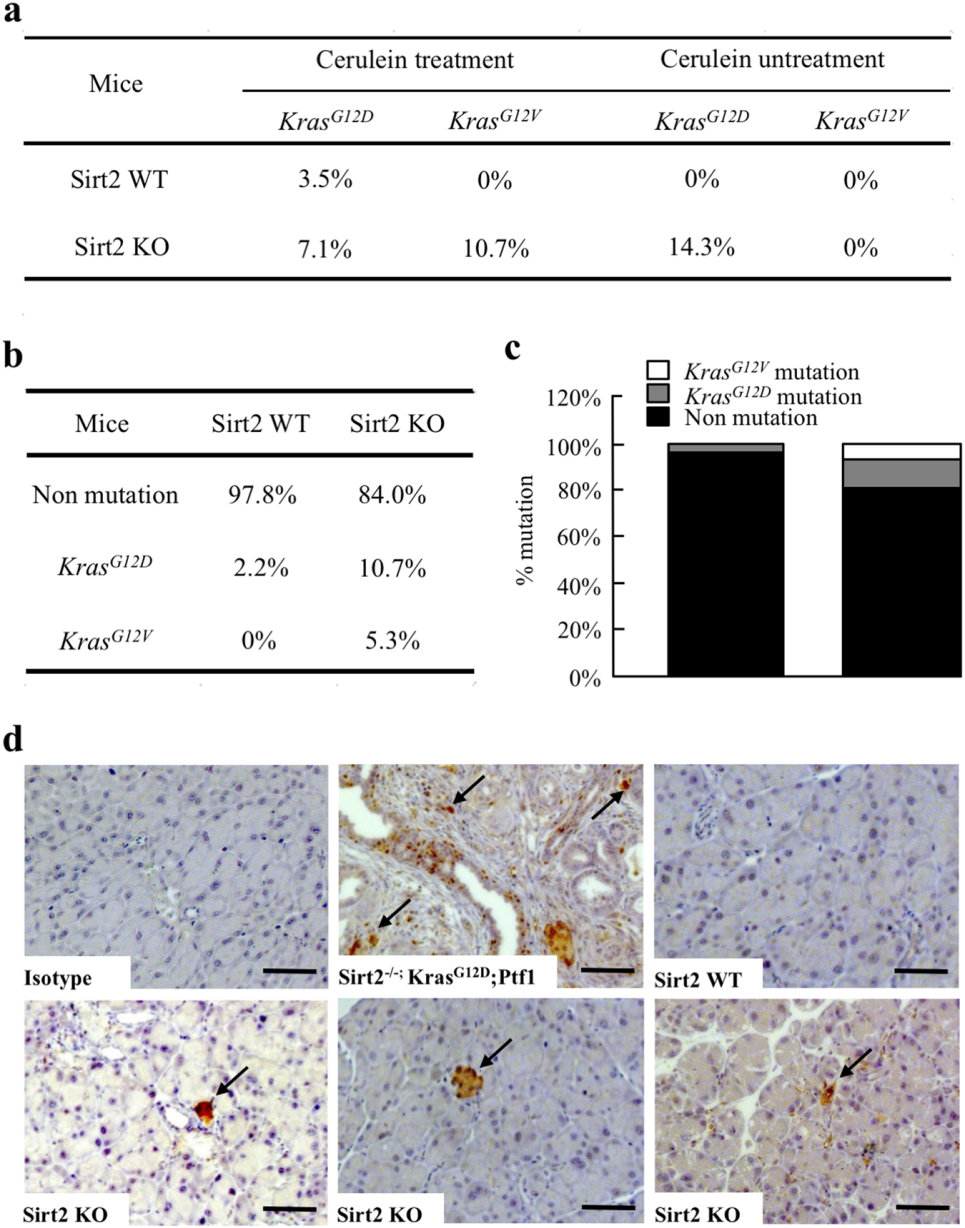
Accumulation of spontaneous tumorigenic Kras mutations in *Sirt2*-deficient mice. **(a)** Accumulation of tumorigenic Kras mutations during inflammation-coupled neoplasm. Mouse pancreas genomic DNA from wild-type and *Sirt2* KO, with and without caerulein-treatment, were analyzed for *Kras*^*G12D*^ or *Kras*^*G12V*^ mutations by competitive allele-specific TaqMan PCR. The table shows the summary of analysis for percentage of mice positive for mutations (46 wild-type mice, 56 *Sirt2* KO mice) after pancreatitis. **(b)** Detail description of wild-type and *Sirt2* KO mice with *Kras*^*G12D*^ and *Kras*^*G12V*^ mutations. **(c)** Bar graph representation of (**b**). (**d**) Immunostaining detection of KRAS-G12D mutant protein in mouse pancreas. Mice pancreas tissues were fixed, embedded in paraffin, and immuno-histochemical staining with anti- KRAS-G12D antibody was performed. Representative images are shown. Bars indicate 100 µm.

To address this question, we have analyzed pancreatic genomic DNA obtained from wild-type and *Sirt2*^−/−^ mice 7 days after caerulein-induced pancreatitis by competitive allele-specific TaqMan Mutation Detection Assays (using castPCR technology) for KRAS amino acid codon mutations at position 12 for glycine to valine (*Kras*^*G12V*^) or aspartic acid (*Kras*^*G12D*^) (Fig. 6). The detection cut-off value was set to 0.1%, as recommended by the manufacturer’s protocol, and pancreas from 46 wild-type (28 treated with caerulein and 18 non-treated) and 56 *Sirt2*^−/−^ (28 treated with caerulein and 28 non-treated) mice were subjected to analysis. A *Kras*^*G12D*^ mutation was detected in 3.5% of the wild-type mouse pancreas at day 7 after caerulein injection, with no *Kras*^*G12V*^ mutations (Fig. 6a, upper column). In contrast, 17.8% of the mice lacking *Sirt2* exhibited a *Kras* mutation (*Kras*^*G12D*^ = 7.1%, *Kras*^*G12V*^ = 10.7%) at day 7 after caerulein exposure (lower column). *Kras* mutations were also observed in untreated *Sirt2*^−/−^ mice (12 to 25-month-old) with 14.3% of *Kras*^*G12D*^ mutation in *Sirt2*^−/−^ mice versus none observed in wild-type mice (Fig. 6a–c). We confirmed *Kras* mutation detection results by TaqMan PCR detection with a completely different design of primer set from Integrated DNA Technologies (IDT) (data not shown). These results indicate that *Sirt2*^−/−^ mice accumulated Kras mutations in the pancreas at a frequency of more than 5 times higher than that of wild-type mice through a caerulein-induced pancreatitis.

To confirm the presence of KRAS-G12D mutant protein, we performed immunohistochemistry with an antibody specific for G12D mutation in mouse KRAS (Fig. 6d). We detected multiple KRAS-G12D mutant protein islands in *Sirt2*^−/−^ but not in wild-type pancreas 7 days after caerulein-induced pancreatitis. These results strongly support our hypothesis that the absence of *Sirt2* provides a growth advantage for cells originally carrying oncogenic *Kras* mutations.

### Identification of differentially expressed genes in S*irt2*^−/−^ mice pancreas on day 2 post caerulein

**Figure 7:**
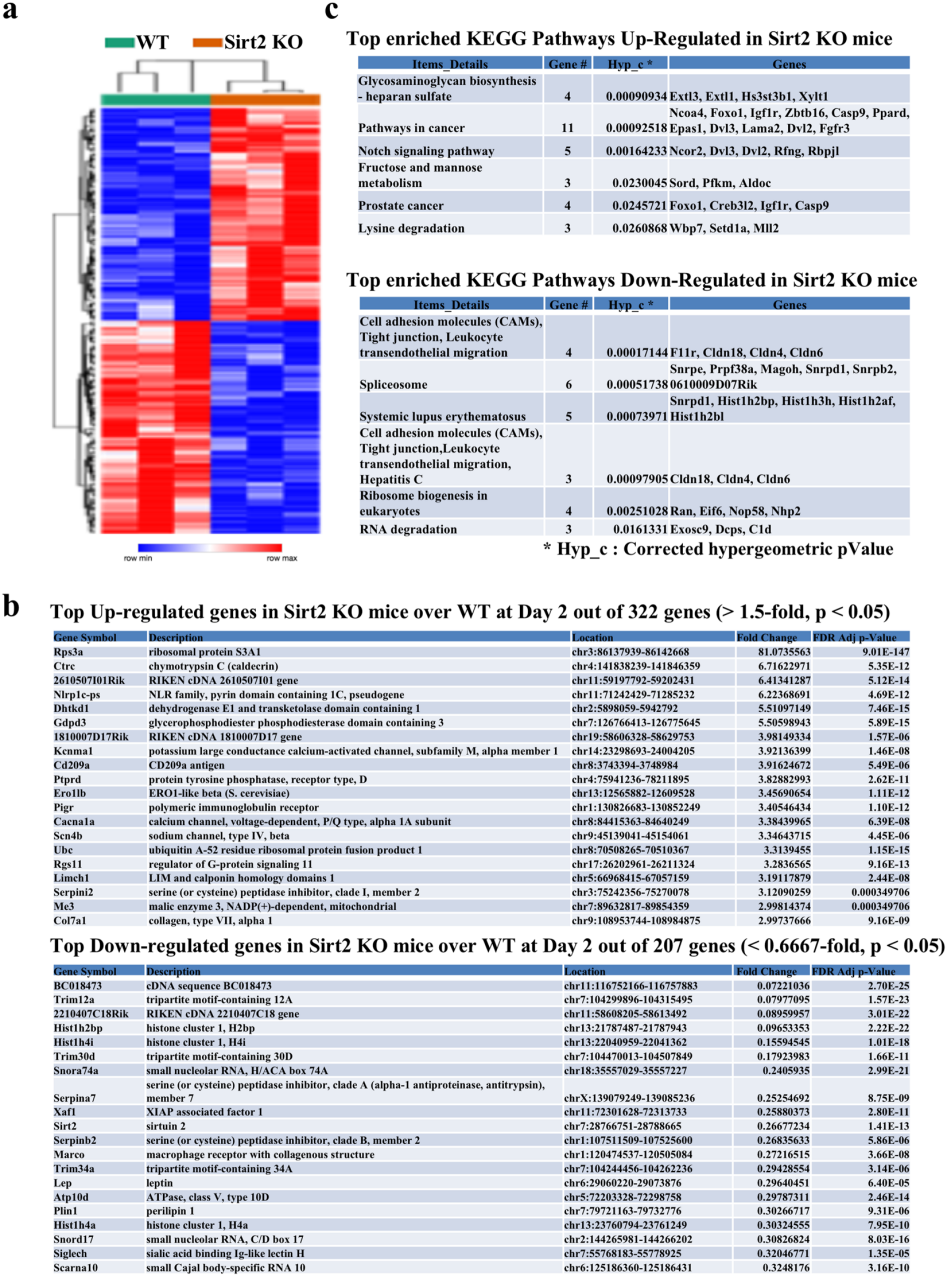
Gene expression profiling of caerulein-induced pancreatitis in wild-type and *Sirt2*-deficient mice. **(a)** The heat map illustrates the expression level of statistically significant 529 differentially expressed genes in pancreas between wild-type and *Sirt2*^−/−^ mice. Each column is a sample, and each row is a gene. The red color represents gene expression greater than the overall mean, and the blue color represents gene expression less than the overall mean. Hierarchical clustering of genes and samples are represented by the dendrograms on the left and across the top of the heat map. **(b)** The list of top 20 genes for the up- and down-regulated differentially expressed genes in *Sirt2*^−/−^ mice pancreas compared with wild-type mice on day 2 post caerulein-induced acute pancreatitis. There are 322 up-regulated and 207 down-regulated statistically significant expressed genes detected. **(c)** KEGG pathway enrichment analysis identified biological pathways associated with the differentially expressed genes detected between wild-type and *Sirt2*^−/−^ mice at 2 days post caerulein-induced pancreatitis. The top 6 enriched up-regulated and down-regulated pathways are listed based on corrected hypergeometric p-value.

Histopathological analyses show that pancreas from mice lacking *Sirt2* exhibited delayed tissue recovery following caerulein exposure. To extend these results at the molecular level and gain insight of a potential mechanism of delayed pancreatic recovery, RNA-seq gene expression profiling was performed. A heat map, from pancreatic RNA in wild-type and *Sirt2*^−/−^ mice, with and without caerulein exposure, on day 2 showed clear differences in expressed genes (Fig. 7a). Overall, we have identified 529 differentially expressed genes (322 up-regulated genes and 207 down-regulated genes, fold-change >1.5, p < 0.05) as part of the top up- and down-regulated genes (Fig. 7b, Supplemental Section, Fig. S9).

The KEGG pathway analysis revealed 17 up-regulated and 9 down-regulated enriched pathways in *Sirt2*^−/−^ mice on day 2 post caerulein-induced pancreatitis (Fig. 7c). Notably, up-regulated pathways included those related to extracellular matrix (ECM) reorganization, cell growth and death, and nutrition regulation, suggesting an active reorganization of ECM due to the continuous cell death and cell growth requiring high nutrition in the *Sirt2*^−/−^ pancreas.

Glycosaminoglycan, including heparin sulfate proteoglycans (HSPGs), biosynthesis is also an enriched pathway in the pancreas of *Sirt2*^−/−^ mice exposed to caerulein. HSPGs are expressed in most cell surfaces and essential for embryogenesis. HSPGs also play important roles in modulating secreted signaling molecules, including growth factors and cytokines^43^.

Aggressive “scrap and build” processes are enriched in “pathways in cancer”. Within this category, there are multiple changes in growth factor receptors’ expression including insulin-like growth factor 1 receptor (*Igf1r*), fibroblast growth factor receptor 3 (*Fgfr3*), and positive regulators of the Ras pathway, such as nuclear receptor coactivator 4 (*Ncoa4*), and segment polarity Wnt pathway proteins dishevelled homolog *Dvl2* and *Dvl3*. Other positive and/or negative cell growth regulatory genes identified include the glucose metabolism regulatory gene *Ncoa4*, peroxisome proliferator-activated receptor gamma (*PPAR*γ), zinc finger, BTB domain-containing protein 16 (*Zbtb16*), and laminin subunit alpha-2 (*Lama2*), an extracellular matrix (EMC) reorganization gene actively involved in cancer development.

The “Lysine degradation” pathway is also enriched in the *Sirt2*^−/−^ mouse, and these genes are involved in pathways that convert lysine to acetyl-coenzyme A (CoA), which is a critical intermediate molecule for lipid metabolism and ATP synthesis, all of which are essential to cell growth and survival. The Ingenuity Pathway Analysis (IPA) also revealed that ECM regeneration was one of the major pathways significantly upregulated in the pancreas from *Sirt2*^−/−^ mice (Supplemental Section, Fig. 9). Importantly, the IPA independently showed that the pancreas from *Sirt2*^−/−^ mice 2 days after exposure exhibit tumorigenic “cell death and survival” genomic profile. Finally, the characterization of these differentially expressed genes is shown. Overall, the transcriptome pathway enrichment analysis supports the notion, at the molecular level, that the regeneration of pancreatic tissue appears to still be occurring at 2 days post pancreatitis induction in *Sirt2*^−/−^ mice.

### Validation of differentially expressed genes at day 2 post pancreatitis induction

**Figure 8:**
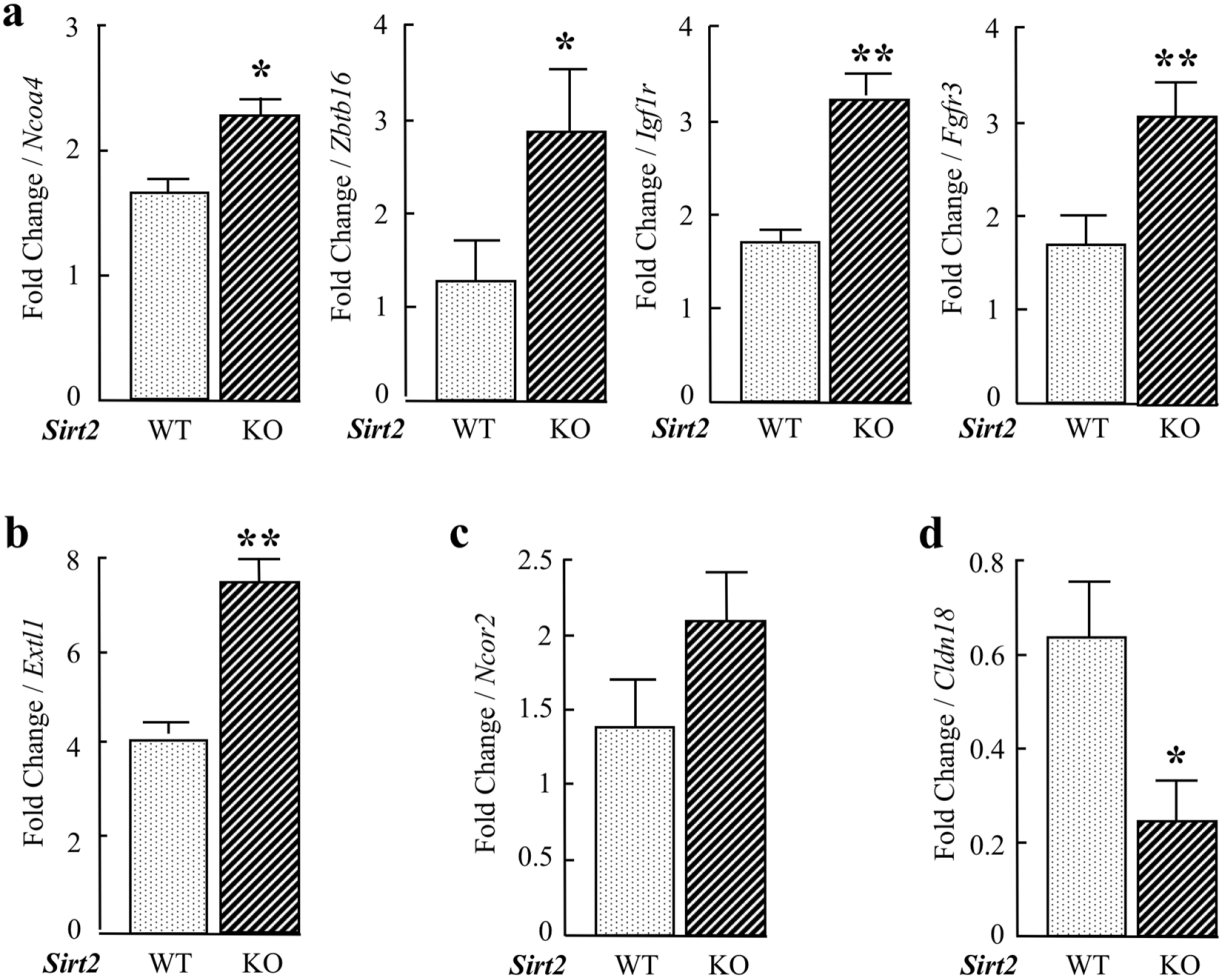
Validation of differentially expressed genes from RNA-seq analysis by RT-qPCR. (**a**) Relative expression levels of *Ncoa4*, *Zbtb16*, *Igf1r*, and *Fgfr3* by RT-qPCR. These genes belong to “Pathways in Cancer”. (**b**) Expression level of *Extl1* that belongs to a pathway “Glycosaminoglycan biosynthesis – heparin sulfate”. (**c**) Expression of *Ncor2*, which belongs to “Notch signaling pathway”. (**d**) Expression of *Cldn18*, which belongs to a pathway of “Cell adhesion molecules (CAMs), Tight junction, Leukocyte transendothelial migration”. Error bars represent ± SEM. Overall, the trend of gene expression by RT-qPCR mirrors that of the RNA-seq analysis. *p < 0.05, **p < 0.01.

To confirm the RNA-seq results, the mRNA levels of *Ncoa4*, *Igf1r*, *Fgfr3*, and *Zbtb16*, which belong to the “Pathways in cancer”, were validated by RT-qPCR, and collectively, the expression of these genes is increased in the *Sirt2*^−/−^ mice (Fig. 8a). In addition, the expression of (Glycosaminoglycan biosynthesis – heparin sulfate) (Fig. 8b), as well as *Ncor2* in “Notch signaling pathway” (Fig. 8c), were also higher in *Sirt2*^−/−^ mice exposed to caerulein, as compared to control mice. In contrast, the expression of *Cldn18* (Cell adhesion molecules, CAMs), is decreased in *Sirt2*^−/−^ mice exposed to caerulein. These results confirm the RNA-seq data, and thus, support the results from the pathway enrichment analysis. We further confirmed differential expression in protein level by immunohistochemical stain for IGF1R and CLDN18 (Supplemental Section, Fig. S8a) and by western blotting for FGFR3 (Supplemental Section, Fig. S8b).

## Discussion

We have reported previously that mice lacking *Sirt2*, which also express a *Kras*^*G12D*^ mutation, develop pancreatic cancer^11^. Acute or chronic inflammation in the pancreas has been demonstrated to be susceptible to oncogenic Kras-mediated transformation^38^, and loss of *Sirt2* leads to a pro-inflammatory phenotype, at least in some part, due to the dysregulation of the acetylation status of the NF-κB subunit^29^. Thus, we proposed that mice lacking *Sirt2* might exhibit a pancreatitis permissive phenotype, as well as *Kras* mutations. In this regard, we have shown that pathologically obvious pancreatic tissue damage was observed at 7 days after the last injection of caerulein, which continued up to at least 6 weeks, suggesting that mice lacking *Sirt2* exhibit a pancreatitis permissive phenotype. In addition, transcriptome analysis revealed differential gene expression patterns, at early time points, indicating that pancreatitis in *Sirt2*^−/−^ mice display enrichment of pathways related to tissue injury and inflammation.

*Kras* mutations are present in the pancreas of healthy individuals^37^, and it seems reasonable to assume that an enhanced inflammatory response or increasing age may play a role, at least in some part, in this process. Caerulein is considered an analogue to cholecystokinin (CCK), a natural hormone synthesized and secreted by enteroendocrine cells in the duodenum, which induces pancreatic enzyme secretion and an inflammatory response^44,45^. Since sirtuins, including SIRT2, play a role both in aging and the immune response, we hypothesized that caerulein exposure might create a mutagenic environment. Our results clearly show that oncogenic *Kras*^*Gly12*^ mutations accumulate after caerulein-induced pancreatitis especially in *Sirt2*^−/−^ mice possibly due to a growth advantage of oncogenic *Kras* mutation. This result is an important evidence to postulate a mechanism of how inflammation contributes to the oncogenic *Kras* mutation initiated pancreatic cancer and show another scientific murine model example of multidimensional interactions between the inflammatory microenvironment and oncogenic *Kras* mutations^40–42^. Recently, it has been reported that inflammation induced *Kras*-independent pancreatic cancers in p53^−/−^ mice^46^. Their results imply that *Kras* mutation is not preferred during tumorigenesis when DNA repair checkpoint is disrupted (p53-null). This could mean that Kras mutations have a potential to escape from p53-dependent genomic integrity severance mechanism or to overcome the elimination pressure by the tumor suppressor function of p53.

Recently, Boggs *et al*. reported transcriptome profiling results by RNA-seq in the recovering pancreas from caerulein-induced pancreatitis^47^. They have acquired transcriptome data at 7 days and 14 days post caerulein-induced pancreatitis, and since the experimental system they used was similar to our study, we have compared their 7 days post pancreatitis expression profiling data with our 2 days post pancreatitis expression profiling data (Supplemental Section, Figs S10 and 11). The heat map depicts a total of 4,102 statistically significant (fold-change >1.5, p-value < 0.05) differentially expressed genes (up-regulated 2066 genes, down-regulated 2036 genes) from our data, which is larger than that of Boggs *et al*. (total 319 genes), possibly due to the date of the analysis. We have focused on the initial stage of pancreatic tissue regeneration to observe the initial difference in recovery pathway between *Sirt2*^−/−^ mice and wild type mice, and Boggs *et al*. analyzed 7 days after pancreatitis in wild type mice when most of the inflammatory response is gone. Indeed, Venn diagrams (Supplemental Section, Fig. S11) revealed that nearly half of the differentially expressed genes identified by Boggs *et al*. overlapped with our data, and a large number of genes are unique for our analysis. Pathway analysis in each category in the Venn diagram (Supplemental Section, Figs S11–13) indicated that the common enriched pathways for both groups related to EMC regeneration, whereas the pathways specifically enriched in ours (Quan *et al*.) are related to cell growth, cell death, and inflammation, supporting the pathological differences in 2 days and 7 days post caerulein-induced pancreatitis that we observed. These results confirmed at the molecular level that 2 days post caerulein-induced pancreatitis is an early time point of pancreatic tissue regeneration.

In closing, our results demonstrate that mice lacking *Sirt2* exhibit a pancreatic inflammation permissive phenotype, as well as accumulate oncogenic *Kras* mutations that is accelerated during recovery from caerulein-induced pancreatitis. Since our previous results have shown that *Sirt2*^−/−^ mice carrying the *Kras*^*G12D*^ mutant progress to pancreatic cancer^11^, there is a reasonable possibility to assume that spontaneous *Kras*^*Gly12*^ mutations accumulated during pancreatitis that could increase the risk of PDAC later in life. Indeed, it has been shown that the level of NAD^+^, an essential substrate for sirtuin activity, declines in the pancreas with age in mice^48^. If the decrease in SIRT2 activity in the pancreas accelerates the accumulation of oncogenic *Kras* mutations, it seems reasonable to propose that caerulein-induced pancreatitis in *Sirt2*^−/−^ mice would be a useful model system for spontaneous *Kras* mutation-initiated pancreatic cancer in humans.

## Methods

### Animals

Acute pancreatitis (AP) was induced into 4-5-month-old female and male *Sirt2* wild-type and knockout mice by 8 hourly intraperitoneal injection of caerulein (100 μg/kg) (Sigma, St. Louis, MO) for one day. Chronic pancreatitis was induced in mice by 6 hourly intraperitoneal injection of caerulein (50 μg/kg) (Sigma, St. Louis, MO) once a week for 6 weeks. Age-matched control mice were administered comparable injections of PBS. For all experiments, age- and sex-matched mice were used, and the same background wild type mice served as controls. Injection procedures were performed as outlined in our murine protocols that have been approved by Northwestern University’s Institutional Animal Care and Use Committee (IACUC, institutional protocol number: 2012–2989). For all experiments, age- and sex-matched mice were used, and the same background wild type mice served as controls. The mice were sacrificed at various time points (day 1, day 2, and day 7) after the last injection, and the tissues were taken for subsequent analyses. Mice were housed and bred in pathogen-free conditions at Northwestern University’s barrier facility with a 12-hour light-dark cycle and could obtain food and water *ad libitum*. All animal housing, handling, and experimental procedures were conducted in strict accordance to the National Institutes of Health and IACUC guidelines. The Northwestern animal resources program is under the direction of full-time veterinarians and is accredited by the Association for Assessment and Accreditation of Laboratory Animal Care. Northwestern complies with the NIH policy on animal welfare, the Animal Welfare Act, and all other applicable federal, state and local laws.

### Histological scoring

Pancreatic tissues were fixed in 10% formalin for 24 hrs at room temperature with slow shaking and embedded in paraffin blocks. Paraffin-embedded tissue samples were cut into 4-μm-thick sections, deparaffinized in xylene, and rehydrated through a graded series of ethanol solutions. To evaluate the pancreatic tissue damage, Hematoxylin-eosin staining was performed. The severity of acute pancreatitis was quantified by assessment of tissue integrity, acinar cell necrosis, and inflammatory cell infiltration. The individual scores and combined scores for these three categories were performed by 3 random images from 5 slides and shown in Supplemental Table 1. To detect tissue fibrosis, the sections were also subjected to Masson’s Trichrome staining. The intensity of stromal fibrosis was scored as: 0: Absent; 1: <5%; 2: 5–10%; 3: 10–20%; 4: >20% fibrosis.

### Immunohistochemistry and immunofluorescence

Pancreatic tissues were fixed in 10% formalin, embedded in paraffin, cut into 4-μm-thick sections. Paraffin sections were deparaffinized in xylene, rehydrated by ethanol gradient, and then heated in a pressure cooker in a citrate-based antigen retrieval buffer. The sections were blocked and incubated with primary antibody α-F4/80 (1:200; Cat. No. ab100790, Abcam, Cambridge, MA) at 4 °C overnight. Subsequently, secondary antibody was added for 30 min, followed by DAB substrate buffer (Abcam, Cambridge, CA) for 5 min. Finally, the sections were counterstained with hematoxylin, dehydrated, and mounted under a coverslip. Immunofluorescence staining was performed according to conventional methods using the following antibodies: α-Amylase (1:200; Cat. No. ab21156, Abcam, Cambridge, MA), α-CK19 (1:200; Cat. No. TROMA-III, University of Iowa Hybridoma Bank), α-PCNA (1:50; Cat. No. sc-56, Santa Cruz Biotech, Santa Cruz, CA) and α-KRAS^G12D^ (1:1000; Cat. No. ab221163, Abcam, Cambridge, MA). AlexaFluro 488 (Cat. No. ab150157, Abcam, Cambridge, MA) and 594 conjugated secondary antibodies (Cat. No. ab150084, Abcam, Cambridge, MA) were used at a 1:200 dilution. The sections were mounted in DAPI containing media (Santa Cruz Biotech, Santa Cruz, CA), exposed to DAPI, FITC, and Texas Red filters, and images were superimposed.

### Flow cytometry and intracellular cytokine staining

Single-cell suspensions from the spleen and pancreas were stained at 4 °C using predetermined optimal concentrations of antibodies for 30 min. Cells with the forward and side scatter properties of lymphocytes were analyzed using Fortessa flow cytometer (BD Bioscience, San Jose, CA). Background staining was assessed using isotype matched control antibodies. The following antibodies were used: FITC-conjugated anti-mouse CD45 (Cat. No. 553079, BD Biosciences, San Jose, CA), PE-conjugated anti-mouse CD11b (Cat. No, 557397, BD Biosciences), APC-conjugated anti-mouse Gr-1 (Ly-6G) (Cat. No. 560599, BD Biosciences), PE-conjugated anti-mouse CD8α (Cat. No. 553032, BD Biosciences), APC-conjugated anti-mouse CD69 (Cat. No. 560689, BD Biosciences), Pacific Blue-conjugated anti-mouse CD4 (Cat. No. 558107, BD Biosciences), FITC-conjugated anti-mouse CD3 (Cat. No. 100203, BD Biosciences, San Jose, CA), APC-conjugated anti-mouse CD25 (Cat. No. 102012, Biolegend, San Diego, CA). For the detection of Tregs, splenocytes were stained with Pacific Blue-conjugated anti-mouse CD4 and APC-conjugated anti-mouse CD25 antibodies, fixed, permeabilized, and subsequently stained with PE-conjugated anti-mouse Foxp3 Ab (Cat. No. 12-5773-82, eBioscience, San Diego, CA).

For intracellular cytokine staining, splenocytes (1 × 10^6^/well in 96 plates) were stimulated at 37 °C in a CO_2_ incubator for 4 hr with Leukocyte Activation Cocktail containing phorbol 12-myristate 13-acetate (PMA), ionomycin, brefeldin A and BD Golgiplug^TM^. This was followed by staining for cell surface CD4 and intracellular interferon-γ (IFN-γ) (Cat. No. 562020, BD Pharmingen, San Diego, CA) or tumor necrosis factor-α (TNF-α) (Cat. No. 561063, BD Pharmingen) using the Intracellular Cytokine Staining Starter Kit (BD Pharmingen). The percentage of IFN-γ and TNF-α producing CD4^+^ T cells was analyzed by Fortessa flow cytometer and FlowJo software.

### Serum amylase activity assay

Fresh blood was collected from experimental mice at various time points (day 1, day 2, and day 7) using a BD Microtainer blood collection tube (BD Biosciences, San Jose, CA) after the last injection. Following centrifugation, serum was kept frozen at −80 °C until assayed. Serum amylase activity was detected with an Amylase Assay Kit (Abcam, Cambridge, MA).

### Identification of Kras mutations

Mutations in *Kras* gene for G12D or G12V were detected by the competitive allele-specific TaqMan PCR in 46 wild-type and 56 *Sirt2*^−/−^ mice. Genomic DNA isolated from mouse pancreas were subjected to TaqMan PCR with custom TaqMan probes specific for mouse *Kras* mutation G12D or G12V (Applied Biosystems). CastPCR Mutation Detection custom made TaqMan probe by Thermo Fisher Scientific (castPCR assay design for Ensemble: ENSMUSG00000030265 Northwestern for mouse KRAS G12V mutation and mouse KRAS G12D mutation custom part number #4476206. Detection cutoff value was set as 0.1%. The CastPCR method was verified using Integrated DNA Technologies (IDT) designed primer set for KRAS G12D GGT/GAT:

KRAS_G12_For: TTTATTGTAAGGCCTGCTGAAA

KRAS_G12_Rev: CTGAATTAGCTGTATCGTCAAGG

KRAS_G12_WT_Allele: TACGCCACCAGC

KRAS_G12D_Allele: TACGCCATCAG

### Next-generation RNA sequencing analysis and bioinformatics

Total RNAs were extracted from pancreatic tissue from PBS or caerulein treated *Sirt2* wild-type and knockout mice using PureLink RNA Mini Kit. The stranded total RNA-seq was conducted in the Northwestern University NUSeq Core Facility. Briefly, total RNA examples were checked for quality on Agilent Bioanalyzer 2100, and quantity with the Qubit fluorometer. The Illumina TruSeq Stranded Total RNA Library Preparation Kit was used to prepare sequencing libraries from 500 ng of total RNA samples. The Kit was performed without modifications. This procedure includes rRNA depletion, remaining RNA purification and fragmentation, cDNA synthesis, 3′ end adenylation, Illumina adapter ligation, library PCR amplification and validation. The lllumina NextSeq. 500 Sequencer was used to sequence the libraries with the production of single-end, 75 bp reads.

### Real-time RT-PCR

Total RNAs were extracted from the pancreatic tissue using PureLink RNA Mini Kit (Invitrogen, Carlsbad, CA). Reverse transcription was performed from 1 μg total RNA using the High-Capacity cDNA Reverse Transcription kit (Applied Biosystems, Foster City, CA), and complementary DNA (cDNA) was used for SYBR green real-time PCR. Amplification was performed with forward and reverse primers using PowerUp^TM^ SYBR Green Master Mix on a QuantStudio^TM^ 7 system (Applied Biosystems, Foster City, CA). The expression of each gene was normalized by corresponding amount of GAPDH mRNA for each condition. Expression of each product were calculated by the comparative ΔΔCt method. Primers are listed in Supplemental Table 2.

### Statistical analysis of expression profiles

The quality of DNA reads, in fastq format, was evaluated using FastQC. Adapters were trimmed, and reads of poor quality or aligning to rRNA sequences were filtered. The cleaned reads were aligned to the *Mus musculus* genome (mm10) using STAR. Read counts for each gene were calculated using htseq-count in conjunction with a gene annotation file for mm10 obtained from UCSC (University of California Santa Cruz; http://genome.ucsc.edu). Normalization and differential expression were determined using DESeq2^49^. The cutoff for determining significantly differentially expressed genes was an FDR-adjusted p-value less than 0.05. Heat map analysis was performed by Morpheus (https://software.broadinstitute.org/morpheus), and Venn diagrams were drawn with BioVenn.

### Pathway enrichment analysis

The enrichment of differentially expressed genes in KEGG pathways were analyzed using GeneCoDis3. Ingenuity Pathway Analysis (IPA) was performed on the differentially expressed genes to identify enriched canonical pathways and gene networks^50^.

### Accession number

GSE115758. The following secure token has been created to allow review of record GSE115758 while it remains in private status: olwlyuqgzxoflql.

### Statistical analysis

Ordinary one-way ANOVAs and two-tailed unpaired t-tests were conducted using GraphPad Prism 6. The statistical significance was reported when p < 0.05.

## Electronic supplementary material

Supplementary Figures

## Data Availability

The datasets generated during the current study are available in the GEO repository. https://www.ncbi.nlm.nih.gov/geo/query/acc.cgi?acc=GSE115758.
