# Supplementary material for: Loss of Sirt2 increases and prolongs a caerulein-induced pancreatitis permissive phenotype and induces spontaneous oncogenic Kras mutations in mice
Source: Sci Rep. 2018 Nov 7;8:16501. doi: 10.1038/s41598-018-34792-y (PMC6220268; doi:10.1038/s41598-018-34792-y)
Supplement: Supplementary file 1 — Supplementary Figures [file 41598_2018_34792_MOESM1_ESM.pdf]

SUPPLEMENTAL SECTION

Loss of *Sirt2* increases and prolongs a cearulein-induced pancreatitis permissive phenotype and induces spontaneous oncogenic Kras mutations in mouse

Songhua Quan[1], Daniel R. Principe[3], Angela E. Dean[1], Seong-Hoon Park[1], Paul J. Grippo[3], David Gius[1,2]*, Nobuo Horikoshi[1]*

[1]Department of Radiation Oncology, [2]Department of Pharmacology, Robert Lurie Cancer Center, Northwestern University Feinberg School of Medicine, Chicago, IL U.S.A.; [3]Department of Medicine, University of Illinois at Chicago, Chicago, IL U.S.A.

Running Title: Loss of Sirt2 increases and prolongs a caerulein-induced pancreatitis

Abstract	178 words
Words	4,826
Figures	8
Pages	29
Characters	33,403
Key words:	SIRT2, Sirtuin, Kras, Mutation, Caerulein, Pancreatitis, Inflammation, RNA-seq, Cancer, Aging

*Co-corresponding Authors:

David Gius, M.D., Ph.D.
Zell Family Scholar Professor
Director, Women's Cancer Research Program
Robert H. Lurie Comprehensive Cancer Center
Vice Chairman Translation Research
Department of Radiation Oncology and Pharmacology
Northwestern University Feinberg School of Medicine
303 East Superior, Rm 4-115
Chicago, IL 60611
312-503-2053
david.gius@northwestern.edu

Nobuo Horikoshi, Ph.D.
Department of Radiation Oncology
Northwestern University Feinberg School of Medicine
303 East Superior, Rm 4-220
Chicago, IL 60611
312-503-0332
nobuo.horikoshi@northwestern.edu

SUPPLEMENTAL SECTION INVENTORY

Figure S1a relates to manuscript Figure 2.
Figure S1b relates to manuscript Figure 2.
Figure S1c relates to manuscript Figure 2.

Figure S2a relates to manuscript Figure 2.
Figure S2b relates to manuscript Figure 2.
Figure S2c relates to manuscript Figure 2.
Figure S2d relates to manuscript Figure 2.

Figure S3a relates to manuscript Figure 2.
Figure S3b relates to manuscript Figure 2.
Figure S3c relates to manuscript Figure 2.

Figure S4a relates to manuscript Figure 2.
Figure S4b relates to manuscript Figure 2.
Figure S4c relates to manuscript Figure 2.

Figure S5a relates to manuscript Figure 5.
Figure S5b relates to manuscript Figure 5.

Figure S6a relates to manuscript Figure 5.
Figure S6b relates to manuscript Figure 5.

Figure S7a relates to manuscript Figure 5.
Figure S7b relates to manuscript Figure 5.

Figure S8a relates to manuscript Figure 8.
Figure S8b relates to manuscript Figure 8.
Figure S8c relates to manuscript Figure 8.

Figure S9 relates to manuscript Figure 7.

Figure S10 relates to manuscript Figure 7.

Figure S11 relates to manuscript Figure 7.

Figure S12 relates to manuscript Figure 7.

Figure S13 relates to manuscript Figure 7.

SUPPLEMENTAL FIGURE

Table 1. Histological scores for acute pancreatitis

Tissue integrity	acinar cell necrosis	inflammatory cell infiltration
0 = absent	0 = no abnormality	0 = no significant infiltration
1 = mild interlobular space	1 = periductal necrosis (<5%)	1 = mild interlobular infiltration
2 = mild interlobular space<br>≥25% peri-parenchyma space	2 = focal necrosis (5-20%)	2 = severe interlobular infiltration<br>≥10% intralobular infiltrate
3 = Moderate interlobular space<br>>25-50% peri-parenchyma space	3 = diffuse parenchymal necrosis (20-50%)	3 = >10-30% intralobular infiltrate
4 = severe interlobular space<br>>50-75% peri-parenchyma space	4 = diffuse parenchymal necrosis (<50-75%)	4 = >30-60% intralobulare infiltrate
5 = severe interlobular space<br>>75-100% peri-parenchyma space	5 = diffuse parenchyma necrosis (>75%)	5 = >60-100% intralobular infiltrate

Table 2. Primers for Quantitative Real-Time PCR

Gene Name	Sequences	
Nuclear receptor coactivator 4 (Ncoa4)	Forward	5′- CTATGGCTCCTGCTAGAATTG -3′
	Reverse	5′- CCAGCTCTCTCCTCTGTATTA -3′
Zbtb16	Forward	5′- TTATGGGAGAGAGGAGAGTG -3′
	Reverse	5′- CACCGAGTAGATACCCAAATG -3′
Insulin-like growth factor 1 receptor (Ifg1r)	Forward	5′- GGAAGGGAACTACTCCTTCTA -3′
	Reverse	5′- ACACACAGCTTGGGATTG -3′
Fibroblast growth factor receptor 3 (Fgfr3)	Forward	5′- TGCTAAATGCCTCCCACGAA -3′
	Reverse	5′- GGGCGAGTCCAATAAGGAGC -3′
Exostosin-like 1 (Extl1))	Forward	5′- GCCATCATTGCTGATGAGA -3′
	Reverse	5′- TCCACCGAGGAGAAGTAAG -3′
Nuclear receptor co-repressor 2 (Ncor2)	Forward	5′- TCTGTCTCCTCAGTACACTC -3′
	Reverse	5′- CTGCCTGTAGCCTCATAATC -3′
Claudin 18 (Cldn18)	Forward	5′- GGTTTGTGGTGTCACTGAT -3′
	Reverse	5′- CATAGCCCTTGGTAGTTGAATA -3′
Platelet-derived growth factor receptor a (Pdfra)	Forward	5′- CGTGCTTGGTCGGATTT -3′
	Reverse	5′- TTCACAGCCACCTTCATTAC -3′
Fc receptor, IgE, High affinity 1, gamma polypeptide (Fcer1g)	Forward	5′- TGGTATTGTCCTTACCCTACT -3′
	Reverse	5′- ACAGCATCTGCTTTCTCAC -3′
C-C motif chemokine ligand 2 (Ccl2)	Forward	5′- GAATGGGTCCAGACATACATTA -3′
	Reverse	5′- TACGGGTCAACTTCACATTC -3′
Transformation related protein 53 (p53)	Forward	5′- CCACCATCCACTACAAGTACA -3′
	Reverse	5′- TCCAGTGTGATGATGGTAAGG -3′

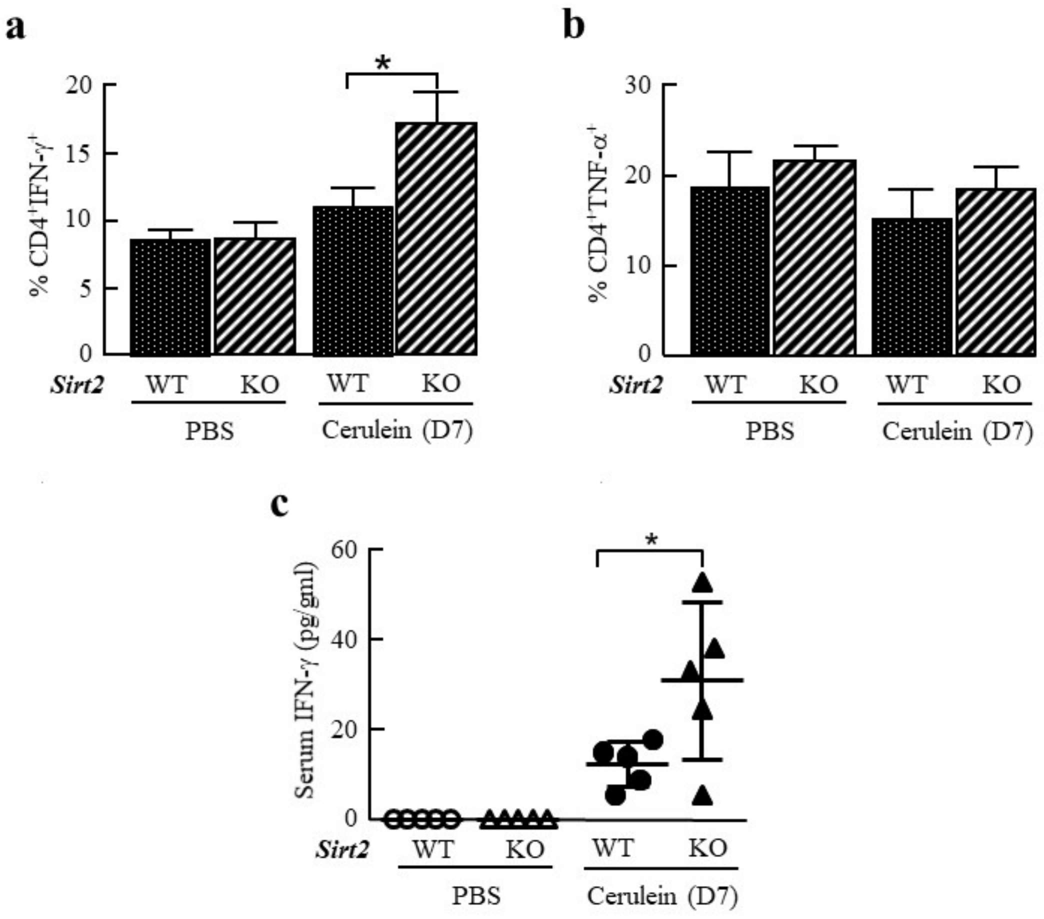

Supplemental Figure S1. (a) IFN-γ producing helper T cell population was determined in wild type and Sirt2$^{-/-}$ mouse spleen 7 days after control (PBS) or caerulein injection. **(b)** TNF-α producing helper T cell population was determined in wild type and Sirt2$^{-/-}$ mouse spleen 7 days after control (PBS) or caerulein injection. **(c)** IFN-γ concentration in serum was determined in wild type and Sirt2$^{-/-}$ mice on 7 days after control (PBS) or caerulein injection.

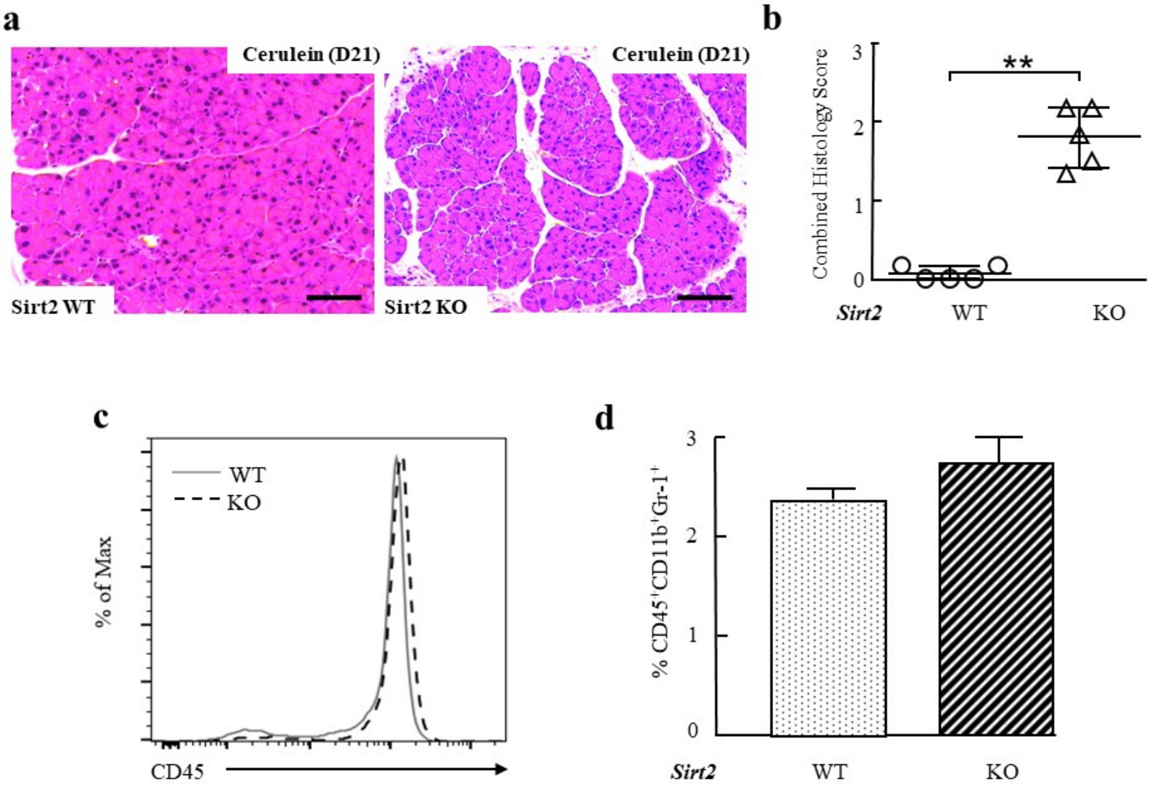

Supplemental Figure S2. Characterization of wild type and Sirt2$^{-/-}$ mice pancreas at 21 days after caerulein-induced pancreatitis. (a) H&E staining of pancreas. **(b)** Histology score of wild type and Sirt2$^{-/-}$ mice pancreas. **(c)** Total infiltrated myeloid cells in pancreas analyzed by CD45 staining. **(d)** Measurement of infiltrated macrophages in wild type and Sirt2$^{-/-}$ mice pancreas. Note that there is a statistically significant increase in infiltrated macrophages in Sirt2$^{-/-}$ mice pancreas.

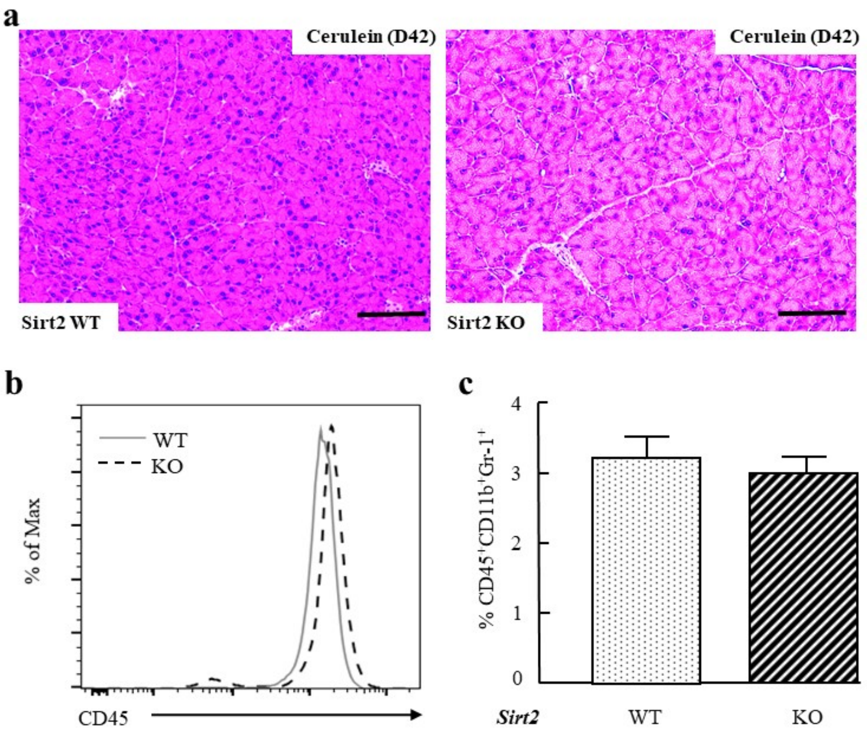

Supplemental Figure S3. Characterization of wild type and Sirt2$^{-/-}$ mice pancreas at 42 days after caerulein-induced pancreatitis. **(a)** H&E staining of pancreas. **(b)** Total infiltrated myeloid cell in pancreas analyzed by CD45 staining. **(c)** Measurement of infiltrated macrophages in wild type and Sirt2$^{-/-}$ mice pancreas.

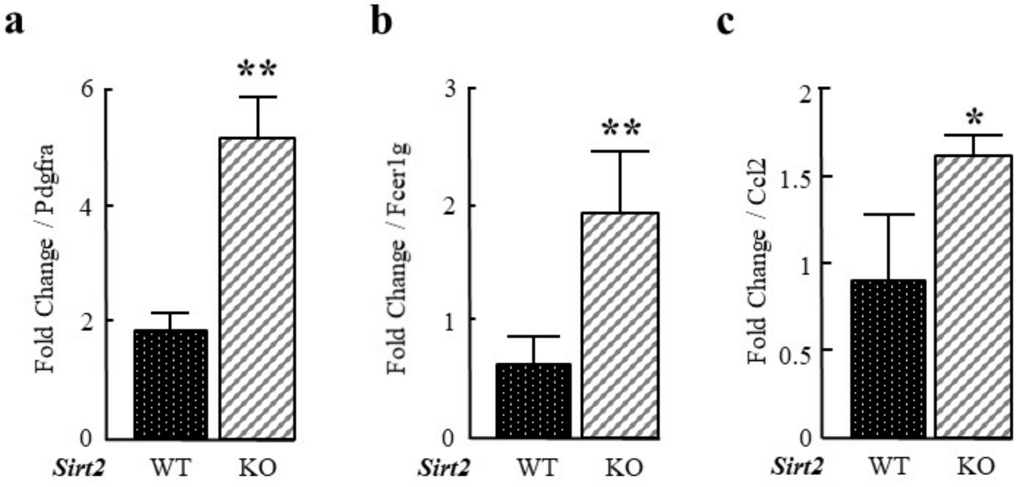

Supplemental Figure S4. The levels of gene expression from NF-kB downstream regulators. Wild-type (WT) and Sirt2$^{-/-}$ (KO) mice were i.p. injected with caerulein, and the pancreas was harvested on day 7 (D7) post-injection. The RNA levels were quantified by RT-PCR, and expression levels for **(a)** Pdgfra, **(b)** Fcer1g, and **(c)** Ccl2 are shown. *$p < 0.05$, **$p < 0.01$.

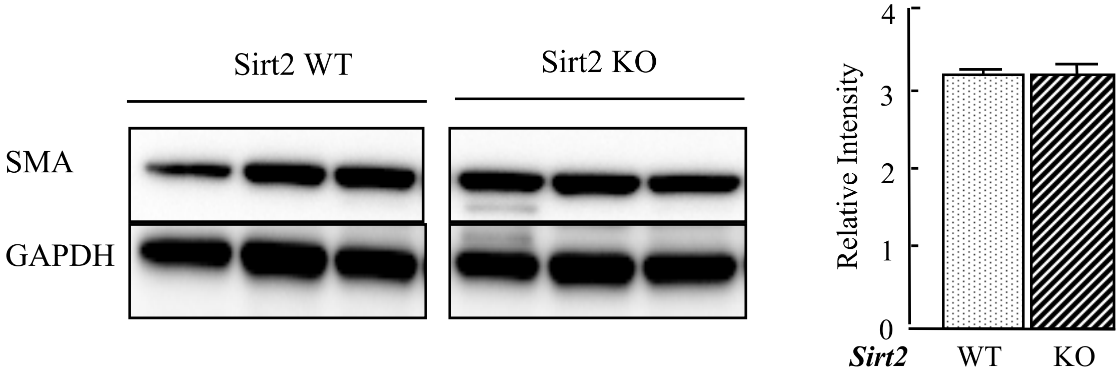

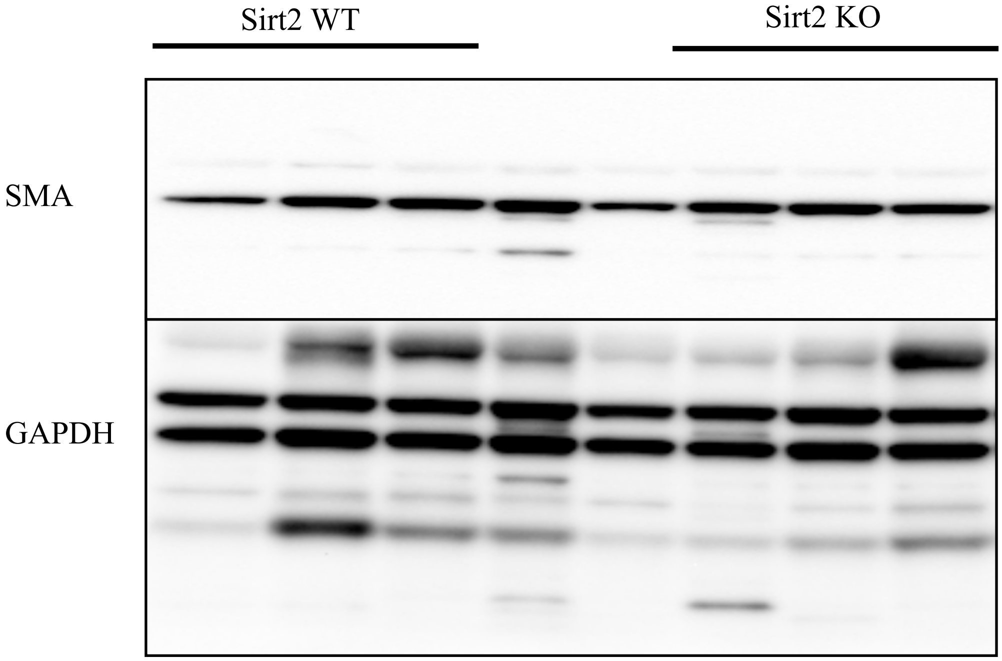

Supplemental Figure S5. Wild-type (WT) and Sirt2$^{-/-}$ (KO) mice were i.p. injected with caerulein, and the pancreas was harvested on day 2 (D2) post-injection. Panels show representative immunoblotting with anti-SMA (Smooth Muscle Actin, Cat. No. ab5694, Abcam, Cambridge, MA) and anti-GAPDH (Proteintech, Rosemont, IL) antibodies (a, left panel) and quantified (a, right panel). (b) Original western blot pictures. For clarification, single exposures of whole blots with continuous lanes are shown in each panel (black edge).

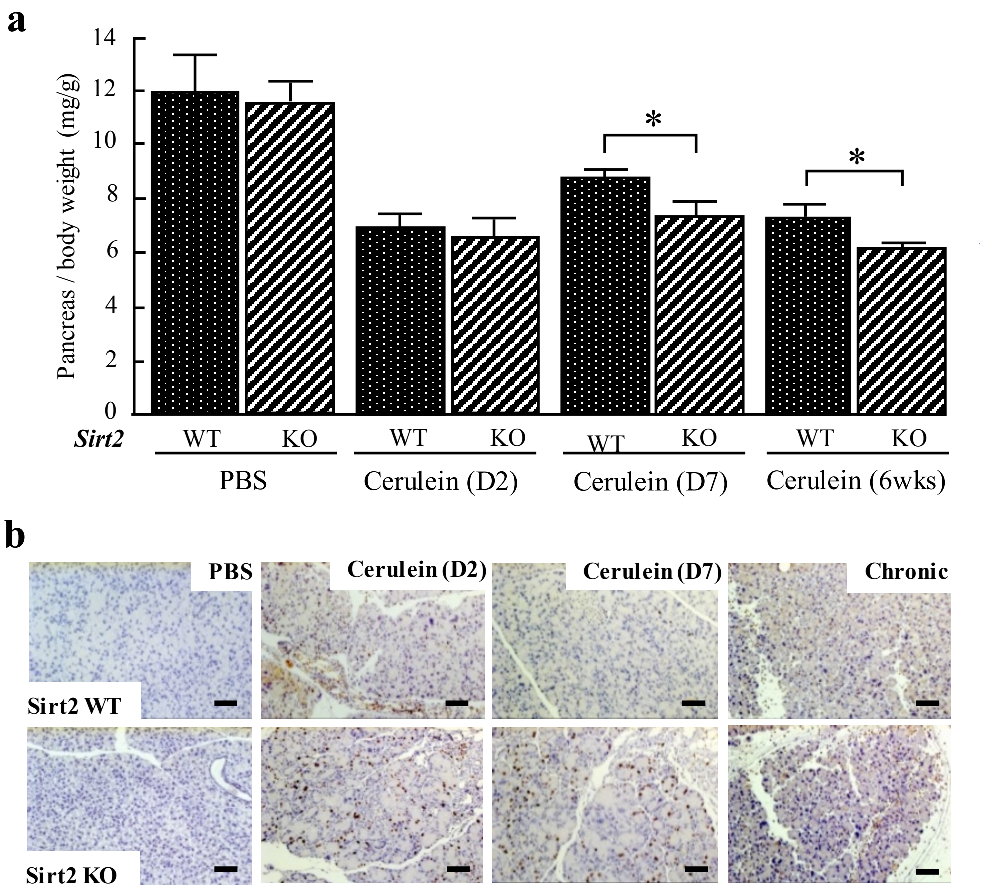

Supplemental Figure S6. (a) Wild-type (WT) and Sirt2$^{-/-}$ (KO) mice were i.p. injected with caerulein, and the pancreas was harvested on day 2 (D2), day 7 (D7), or 6 weeks post-injection. Mouse pancreas weight and body weight ratio is shown. **(b)** The pancreas tissues were fixed, embedded in paraffin, and stained with Ki67 antibody (1:100; Cat. No. ab16667, Abcam, Cambridge, MA). Panels show representative immuno-histochemical staining of Ki67 antibody. Bars indicate 100 μm. (n=5, *p < 0.05).

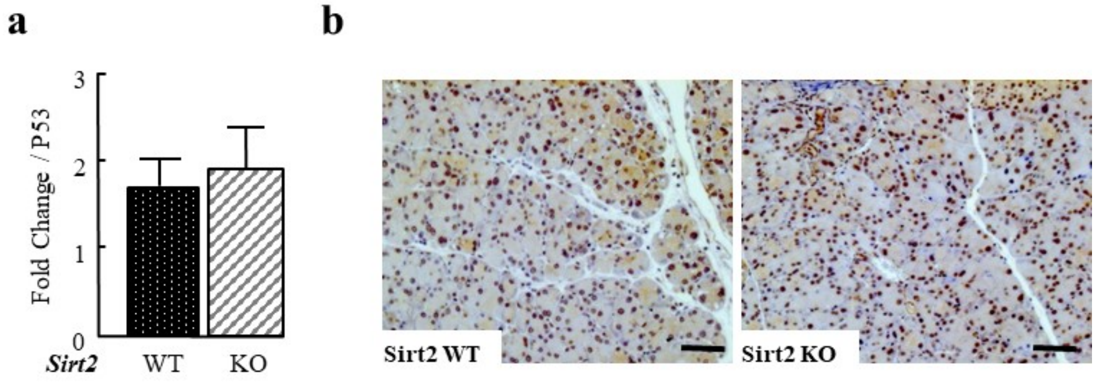

Supplemental Figure S7. Wild-type (WT) and Sirt2$^{-/-}$ (KO) mice were i.p. injected with caerulein, and the pancreas was harvested on day 7 (D7) post-injection. **(a)** The mRNA levels for the p53 were quantified by RT-qPCR. **(b)** Panels show representative immuno-histochemical staining of p53 antibody (1:200; Cat. No. ab131442, Abcam, Cambridge, MA). Bars indicate 100 μm.

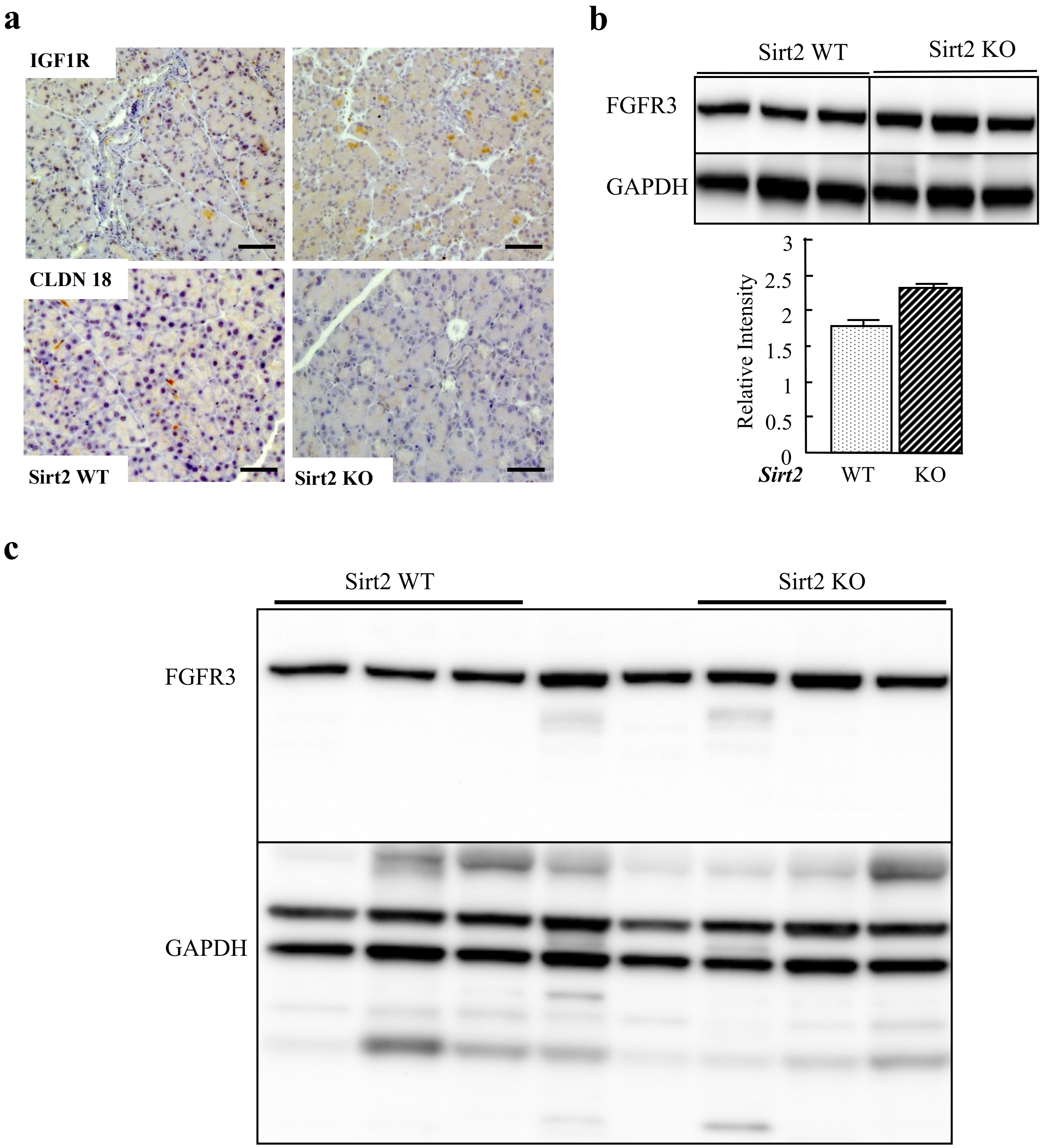

Supplemental Figure S8. (a) Wild-type (WT) and Sirt2$^{-/-}$ (KO) mice were i.p. injected with caerulein, and the pancreas was harvested on day 2 (D2) post-injection. Panels show representative immuno-histochemical staining with anti-IGF1R (1:200; left-upper panel; Cat. No. ab39675, Abcam, Cambridge, MA) and anti-CLDN18 (1:400; left-bottom panel; Cat. No. 21126-AP, Proteintech, Rosemont, IL) antibodies. Bars indicate 100 µm. **(b)** Panels show representative immunoblotting with anti-FGFR3 (Cat. No. ab133644, Abcam, Cambridge, MA) and anti-GAPDH (Cat. No. 60004-1-Ig, Proteintech, Rosemont, IL) antibodies (right-upper panel) and were quantified (right-bottom panel). (c) Original western blot pictures. For clarification, single exposures of whole blots with continuous lanes are shown in each panel (black edge).

Ingenuity Pathway Analysis

Top Canonical Pathways

Name	p-value	Overlap	
DNA Methylation and Transcriptional Repression Signaling	1.72E-06	26.5 %	9/34
GP6 Signaling Pathway	1.77E-05	11.9 %	16/134
Hepatic Fibrosis / Hepatic Stellate Cell Activation	3.07E-05	10.2 %	19/187
TR/RXR Activation	3.51E-05	13.3 %	13/98
Cardiac Hypertrophy Signaling	8.52E-05	8.9 %	21/236

Top Upstream Regulators

Upstream Regulator	p-value of overlap	Predicted Activation
TP53	3.72E-17	
dexamethasone	1.02E-16	
ERBB2	2.40E-11	
TCF3	2.57E-11	
TGFB1	2.63E-10	

Top Diseases and Bio Functions

Diseases and Disorders

Name	p-value	#Molecules
Cancer	2.07E-05 - 3.95E-23	704
Organismal Injury and Abnormalities	2.07E-05 - 3.95E-23	722
Gastrointestinal Disease	2.00E-05 - 1.20E-22	663
Dermatological Diseases and Conditions	9.82E-06 - 1.23E-20	463
Reproductive System Disease	1.91E-05 - 2.52E-20	489

Molecular and Cellular Functions

Name	p-value	#Molecules
Cell Death and Survival	2.05E-05 - 4.31E-18	317
Cellular Movement	1.66E-05 - 1.35E-16	218
Post-Translational Modification	2.14E-07 - 1.37E-14	88
Protein Synthesis	9.92E-06 - 1.37E-14	107
Protein Trafficking	2.00E-09 - 1.37E-14	37

Supplemental Figure S9. Ingenuity Pathway Analysis of 529 differentially expressed genes detected between wild type and Sirt2$^{-/-}$ mice pancreas on day2 post caerulein-induced pancreatitis.

Up-regulated genes

Gene Symbol	Description	Location	Log2 Fold Change	FDR Adj p-Value
Reg3g	regenerating islet-derived 3 gamma	chr6:78466268-78468874	7.12569395	2.04E-73
Gm2663	predicted gene 2663	chr6:40995821-40999479	9.61396035	9.13E-56
Spp1	secreted phosphoprotein 1	chr5:104436191-104441053	4.18976183	1.08E-47
1810009J06Rik	RIKEN cDNA 1810009J06 gene	chr6:40964771-40968427	7.81480833	2.87E-40
Ms4a6d	membrane-spanning 4-domains, subfamily A, member 6D	chr19:11586605-11604804	5.92156317	3.28E-38
Tlr13	toll-like receptor 13	chrX:106143274-106160493	5.44833132	1.33E-36
Serpina10	serine (or cysteine) peptidase inhibitor, clade A (alpha-1 antiproteinase, antitrypsin), member 10	chr12:103616674-103631444	3.93155526	1.71E-29
Ms4a7	membrane-spanning 4-domains, subfamily A, member 7	chr19:11321038-11334695	5.77986679	2.80E-28
Cilp	cartilage intermediate layer protein, nucleotide pyrophosphohydrolase	chr9:65265179-65280605	4.6081288	2.96E-21
Gpnmb	glycoprotein (transmembrane) nmb	chr6:49036517-49058182	5.69847307	1.14E-20
S100a4	S100 calcium binding protein A4	chr3:90603769-90606045	4.70733207	1.14E-20
Lgals3	lectin, galactose binding, soluble 3	chr14:47373859-47386167	4.69026894	1.37E-19
Arhgap19	Rho GTPase activating protein 19	chr19:41766587-41802084	4.2064501	4.19E-18
C3ar1	complement component 3a receptor 1	chr6:122847139-122856157	4.55582776	6.34E-18
Postn	periostin, osteoblast specific factor	chr3:54361106-54391041	4.50532642	2.20E-16
Ccr2	chemokine (C-C motif) receptor 2	chr9:124102182-124109140	4.84927328	1.03E-15
Alox5ap	arachidonate 5-lipoxygenase activating protein	chr5:149265003-149288153	3.8164267	1.34E-15
Lgmn	legumain	chr12:102394081-102439813	4.62222774	4.52E-15
Ms4a6c	membrane-spanning 4-domains, subfamily A, member 6C	chr19:11469367-11482196	4.15097987	5.67E-15
Hpgds	hematopoietic prostaglandin D synthase	chr6:65117292-65144730	3.4092984	2.72E-14

Down-regulated genes

Gene Symbol	Description	Location	Log2 Fold Change	FDR Adj p-Value
Ctrc	chymotrypsin C (caldecrin)	chr4:141838239-141846359	-3.9360604	7.96E-16
Pnlip	pancreatic lipase	chr19:58670364-58681788	-4.5575342	1.26E-15
Rn45s	45S pre-ribosomal RNA	chr17:39842996-39848829	-3.02967	6.22E-14
Ranbp3l	RAN binding protein 3-like	chr15:8967948-9067333	-4.195986	1.71E-13
Ltbp3	latent transforming growth factor beta binding protein 3	chr19:5740903-5758532	-2.3565848	2.40E-13
Lars2	leucyl-tRNA synthetase, mitochondrial	chr9:123366939-123462664	-3.8203953	2.82E-13
Fgf21	fibroblast growth factor 21	chr7:45613889-45615490	-3.0990804	3.64E-13
Cela1	chymotrypsin-like elastase family, member 1	chr15:100674421-100687920	-4.0376911	6.84E-12
Wwc1	WW, C2 and coiled-coil domain containing 1	chr11:35839177-35980089	-2.1119957	7.51E-11
Celsr2	cadherin, EGF LAG seven-pass G-type receptor 2	chr3:108390847-108415494	-2.9503111	8.01E-11
Ttyh1	tweety family member 1	chr7:4119529-4135407	-2.704502	9.10E-11
Padi2	peptidyl arginine deiminase, type II	chr4:140906359-140952586	-2.6832618	1.71E-10
Clps	colipase, pancreatic	chr17:28558209-28560766	-3.5045198	3.50E-10
Hamp2	hepcidin antimicrobial peptide 2	chr7:30922371-30924181	-4.0437466	5.31E-10
Cpa1	carboxypeptidase A1, pancreatic	chr6:30639217-30645361	-3.2531244	5.60E-10
Shank1	SH3/ankyrin domain gene 1	chr7:44310263-44358353	-4.3252635	6.27E-10
2210010C04Rik	RIKEN cDNA 2210010C04 gene	chr6:41030267-41035509	-3.5962821	6.67E-10
Ctrl	chymotrypsin-like	chr8:105931993-105933862	-3.587345	2.14E-09
Mat1a	methionine adenosyltransferase I, alpha	chr14:41105032-41124428	-2.77516	2.83E-09
Cryab	crystallin, alpha B	chr9:50752970-50756635	-1.9058018	5.97E-09

Supplemental Figure S10. Top 20 up- and down-regulated statistically significant differentially expressed genes between control (PBS) and caerulein injected wild type mice pancreas at 2 days post pancreatitis induction. There are 2066 up-regulated and 2036 down regulated genes (fold change > 1.5, $p < 0.05$) identified (total 4102 genes). Ranking is based on FDR adjusted p-value of each gene.

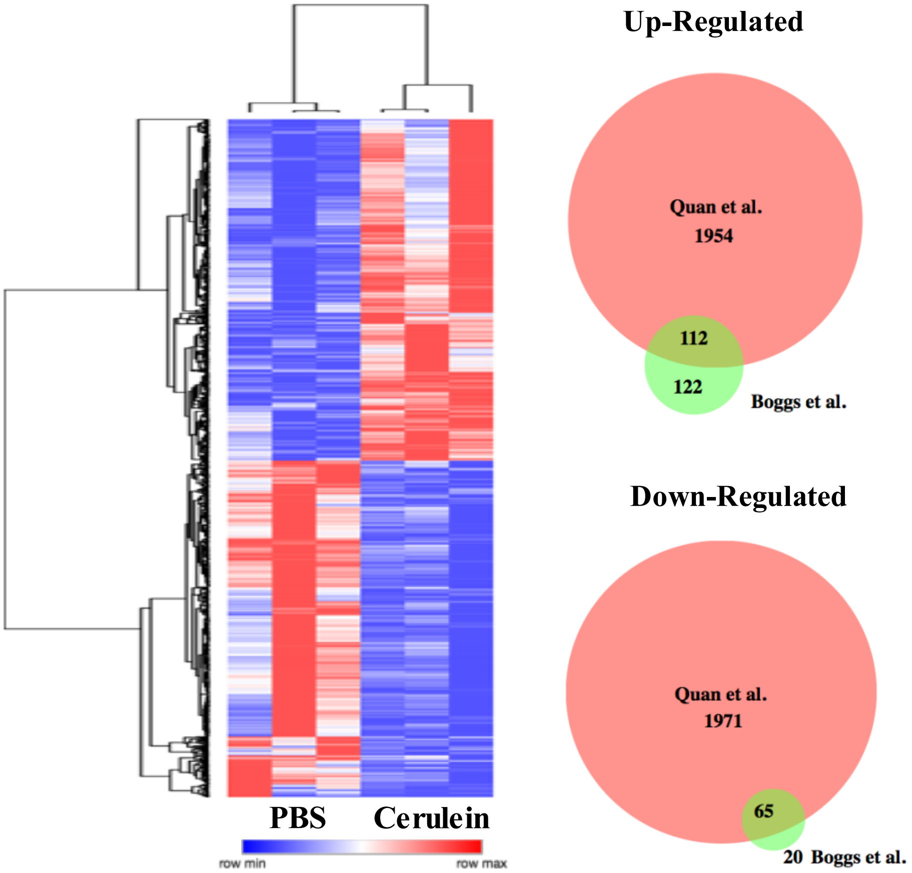

Supplemental Figure S11. Characterization of differentially expressed genes. Heat map depicts all differentially expressed genes at a total of 4102 genes. Venn diagrams indicate the relationship of up- or down-regulated genes identified between Boggs et al. (Boggs et al., 2018) and Quan et al. (current study). Numbers in the Venn diagrams show the number of genes in each category.

Up-Regulated

Quan *et al.* AND Boggs *et al.* (112)

Items	Items_Details	Support	Hyp_c	Genes
Kegg:04974,Kegg:04510,Kegg:04512	Protein digestion and absorption,Focal adhesion,ECM-receptor interaction	6	2.69E-10	Col6a3,Col6a1,Col5a2,Col3a1,Col5a1,Col1a2
Kegg:04974	Protein digestion and absorption	8	7.92E-10	Col6a3,Col6a1,Col14a1,Slc16a10,Col5a2,Col3a1,Col5a1,Col1a2
Kegg:04512	ECM-receptor interaction	8	1.17E-09	Col6a3,Spp1,Col6a1,Col5a2,Col3a1,Col5a1,Col1a2,Cd44
Kegg:04510,Kegg:04512	Focal adhesion,ECM-receptor interaction	7	4.85E-09	Col6a3,Spp1,Col6a1,Col5a2,Col3a1,Col5a1,Col1a2
Kegg:04610	Complement and coagulation cascades	7	1.06E-08	C1qa,C3ar1,C1qc,Pros1,Plau,F3,C1qb
Kegg:04510	Focal adhesion	9	2.60E-08	Pdgfra,Col6a3,Spp1,Col6a1,Col5a2,Col3a1,Col5a1,Col1a2,Hgf

Quan *et al.* *ONLY* (1954)

Items	Items_Details	Support	Hyp_c	Genes
Kegg:04110	Cell cycle	48	1.16E-27	Cdkn2b,Anapc10,Ccnb1,Cdk2,Mad2l1,Trp53,Cdk1,Ywhaq,Orc1,Chek2,Cdc14b,Mcm3,Ccna2,Pcna,Gadd45a,Espl1,Anapc4,Bub1,Skp2,Dbf4,Gadd45b,Mdm2,Orc6,Orc2,Mcm4,Ccne2,Bub3,Mad2l2,Chek1,Ywhah,Mcm6,Atr,Cdc25b,Cdkn1a,Cdc20,Bub1b,Cdc6,Ccnb2,Cdk4,Cdc45,Anapc7,Cdc25c,Orc5,Plk1,Mcm7,Mcm5,Ttk,Tgfb1
Kegg:03030	DNA replication	22	2.78E-18	Ssbp1,Mcm3,Pcna,Pola1,Pold3,Prim2,Pole,Rfc5,Prim1,Mcm4,Rfc2,Lig1,Dna2,Mcm6,Pold4,Rfc4,Rnaseh2b,Rfc3,Mcm7,Pole3,Mcm5,Pole2
Kegg:04142	Lysosome	38	3.55E-18	Laptm4a,Galc,Ctsc,Ap1s2,Ctsa,Ctsl,Galns,Hexa,Tcirg1,Gusb,M6pr,Hexb,Ctsk,Manba,Npc2,Lipa,Psap,Gla,Ctss,Clta,Gns,Cltc,Ctsd,Lamp2,Dnase2a,Pla2g15,Npc1,Ctsz,Acp2,Ap1s1,Cd68,Ppt1,Asah1,Ctsb,Slc11a1,Fuca1,Scarb2,Ctsh
Kegg:04380	Osteoclast differentiation	36	2.89E-17	Fcgr1,Pik3r5,Tgfbr1,Lilrb3,Nfkbia,Trem2,Ncf2,Ctsk,Tec,Irf9,Fcgr2b,Btk,Fhl2,Lilra6,Nfkb2,Junb,Pik3cg,Fcgr4,Pik3r3,Cyba,Ncf4,Sqstm1,Tyrobp,Tnfrsf11b,Sirpa,Ifnar2,Jun,Csf1r,Ifnar1,Ncf1,Ifngr2,Socs3,Cybb,Tgfb1,Sfpi1,Ifngr1
Kegg:04145	Phagosome	40	2.69E-15	Fcgr1,Stx7,Tuba1b,Thbs4,Tubb2a,Ctsl,Ncf2,Tuba1a,Actg1,Tcirg1,Itgam,M6pr,Dync1i2,Tubb6,Fcgr2b,Tlr2,Ctss,Itgb1,Cd14,Atp6v0e,Mrc2,Atp6v1g1,Atp6v1b2,Itgb5,Tubb5,Lamp2,Rab5c,Fcgr4,Sftpd,Thbs2,Atp6v1a,Cyba,Ncf4,Itga2,Tuba1c,Ncf1,Itgav,Cybb,5430435G22Rik,Thbs1
Kegg:04115	p53 signaling pathway	25	3.21E-14	Lrdd,Casp8,Ccnb1,Cdk2,Trp53,Cdk1,Chek2,Gadd45a,Serpine1,Gadd45b,Apaf1,Mdm2,Bid,Ccne2,Chek1,Casp3,Atr,Ccng2,Ccng1,Cdkn1a,Gtse1,Ccnb2,Cdk4,Bax,Thbs1

Boggs *et al.* *ONLY* (122)

Items	Items_Details	Support	Hyp_c	Genes
Kegg:04950	Maturity onset diabetes of the young	5	7.04E-08	Neurod1,Ins1,Iapp,Ins2,Slc2a2
Kegg:04930,Kegg:04950	Type II diabetes mellitus,Maturity onset diabetes of the young	3	2.63E-06	Ins1,Ins2,Slc2a2
Kegg:04810,Kegg:05215	Regulation of actin cytoskeleton,Prostate cancer	3	0.00025903	Ins1,Pdgfc,Ins2
Kegg:04940	Type I diabetes mellitus	3	0.00089311	Cpe,Ins1,Ins2
Kegg:04810	Regulation of actin cytoskeleton	4	0.00432964	Fgf12,Ins1,Pdgfc,Ins2
Kegg:04010	MAPK signaling pathway	4	0.0077085	Ntrk2,Fgf12,Il1r1,Pla2g4a

(Hyp_c: Corrected hypergeometric pValue)

Supplemental Figure S12. KEGG pathway enrichment analysis of up-regulated genes from the Venn diagrams in Suppl. Figure 8. Within the up-regulated genes, the common genes (112 genes) for both Boggs et al.(Boggs et al., 2018) and Quan et al. (current study), unique in Quan et al. (1954 genes), and unique in Boggs et al. (122 genes) were analyzed for pathway enrichment.

Down-Regulated

Quan *et al.* AND Boggs *et al.* (65)

Items	Items_Details	Support	Hyp_c	Genes
Kegg:04974,Kegg:04972	Protein digestion and absorption,Pancreatic secretion	6	2.50E-12	Prss2,Cela3b,Ctrb1,Ctrl,Cpa1,2210010C04Rik
Kegg:04972	Pancreatic secretion	7	3.94E-10	Prss2,Cela3b,Ctrb1,Pnlip,Ctrl,Cpa1,2210010C04Rik

Quan *et al. ONLY* (1971)

Items	Items_Details	Support	Hyp_c	Genes
Kegg:04210,Kegg:04910	Apoptosis,Insulin signaling pathway	4	0.0302041	Prkaca,Prkar2a,Pik3r1,Akt1
Kegg:04210,Kegg:04910,Kegg:04062,Kegg:04914	Apoptosis,Insulin signaling pathway,Chemokine signaling pathway,Progesterone-mediated oocyte maturation	3	0.0472238	Prkaca,Pik3r1,Akt1
Kegg:04210,Kegg:05200,Kegg:05145,Kegg:05212,Kegg:05215,Kegg:05210,Kegg:05213,Kegg:04370,Kegg:05222,Kegg:05223	Apoptosis,Pathways in cancer,Toxoplasmosis,Pancreatic cancer,Prostate cancer,Colorectal cancer,Endometrial cancer,VEGF signaling pathway,Small cell lung cancer,Non-small cell lung cancer	3	0.0343563	Casp9,Pik3r1,Akt1
Kegg:04910	Insulin signaling pathway	28	3.00E-08	Prkcz,Mknk1,Pde3b,Slc2a4,Ppargc1a,Prkaca,Foxo1,Insr,Sos2,Acaca,Prkaa2,Acacb,Srebf1,Prkar2a,Rps6kb1,Rps6kb2,Mtor,Phka1,Lipe,Pik3r1,Sorbs1,Rptor,Mapk9,Fasn,Pklr,Irs1,Akt1,Tsc2
Kegg:04910,Kegg:05160,Kegg:04010,Kegg:04722,Kegg:05200,Kegg:04510,Kegg:04012,Kegg:04660,Kegg:04664	Insulin signaling pathway,Hepatitis C,MAPK signaling pathway,Neurotrophin signaling pathway,Pathways in cancer,Focal adhesion,ErbB signaling pathway,T cell receptor signaling pathway,Fc epsilon RI signaling pathway	3	0.0289007	Sos2,Mapk9,Akt1
Kegg:04910,Kegg:05160,Kegg:04722,Kegg:05200,Kegg:04062,Kegg:04630,Kegg:04510,Kegg:05211,Kegg:05214,Kegg:05215,Kegg:05221,Kegg:05213,Kegg:04012,Kegg:04660,Kegg:04662,Kegg:04664,Kegg:05220,Kegg:05223	Insulin signaling pathway,Hepatitis C,Neurotrophin signaling pathway,Pathways in cancer,Chemokine signaling pathway,Jak-STAT signaling pathway,Focal adhesion,Renal cell carcinoma,Glioma,Prostate cancer,Acute myeloid leukemia,Endometrial cancer,ErbB signaling pathway,T cell receptor signaling pathway,B cell receptor signaling pathway,Fc epsilon RI signaling pathway,Chronic myeloid leukemia,Non-small cell lung cancer	3	0.0472238	Sos2,Pik3r1,Akt1

(Hyp_c: Corrected hypergeometric pValue)

Boggs *et al. ONLY* (20)

There are not annotations significantly enriched.

Supplemental Figure S13. KEGG pathway enrichment analysis of down-regulated genes from the Venn diagrams in Suppl. Figure 8. Within the down-regulated genes, the common genes (65 genes) for both Boggs et al.(Boggs et al., 2018) and Quan et al. (current study), unique in Quan et al. (1971 genes), and unique in Boggs et al. (20 genes) were analyzed for pathway enrichment. For the genes unique in Boggs et al., there were no significantly enriched pathways identified.
